# Identifying inflammatory bowel disease subtypes: a comprehensive exploration of transcriptomic data and machine learning-based approaches

**DOI:** 10.1177/17562848251362391

**Published:** 2025-08-12

**Authors:** Niyati Saini, Animesh Acharjee

**Affiliations:** Cancer and Genomic Sciences, School of Medical Sciences, College of Medicine and Health, University of Birmingham, Birmingham, UK; Cancer and Genomic Sciences, School of Medical Sciences, College of Medicine and Health, University of Birmingham, Birmingham B15 2TT, UK; Institute of Translational Medicine, University Hospitals Birmingham NHS Foundation Trust, Birmingham B15 2TT, UK; Centre for Health Data Research, University of Birmingham, Birmingham B15 2TT, UK

**Keywords:** Crohn’s disease, IBD subtypes, machine learning, transcriptomics, ulcerative colitis

## Abstract

**Background:**

Inflammatory bowel disease (IBD), encompassing Crohn’s disease (CD) and ulcerative colitis (UC), is a heterogeneous condition characterised by chronic gastrointestinal inflammation and dysregulated immune responses. Despite advances in transcriptomic analysis and machine learning (ML), consistent molecular subtyping across datasets remains a challenge. There is a critical need for robust subtypes that reflect disease heterogeneity and correlate with clinical outcomes.

**Objectives:**

Unlike prior studies focused on either UC or CD or based on small datasets, this study analyses a large-scale RNA sequencing (RNA-seq) dataset to identify transcriptomic subtypes in both UC and CD.

**Design:**

We analysed RNA-seq data from four prospective cross-sectional cohorts from Gene Expression Omnibus: GSE193677, GSE186507, GSE137344 and GSE235236.

**Methods:**

Analysed RNA-sequenced data from inflamed and non-inflamed intestinal biopsies of 2490 adult IBD patients. *K*-means clustering was applied independently to UC and CD samples to identify transcriptomic clusters. Gene set enrichment and network analyses explored molecular characteristics. Associations with clinical metadata, including disease severity and anatomical involvement, were assessed using Chi-square and analysis of variance tests.

**Results:**

*K*-means clustering revealed three distinct transcriptomic subtypes in both UC and CD. In UC, Cluster 1 was enriched for RNA processing and DNA repair genes; Cluster 2 highlighted autophagy, stress responses and upregulation of *ATG13, VPS37C* and *DVL2*; Cluster 3 emphasised cytoskeletal organisation (*SRF, SRC* and *ABL1*). In CD, Cluster 1 featured cytoskeletal remodelling and suppressed protein synthesis (*CFL1, F11R* and *RAD23A*), while Cluster 2 upregulated stress and translation pathways. Cluster 3 again prioritised cytoskeletal structure over metabolic activity. Cluster 3 in both conditions was significantly associated with moderate-to-severe endoscopic activity; Cluster 1 was enriched in inactive or mild disease.

**Conclusion:**

We report three transcriptomic subtypes in UC and CD, each with distinct molecular signatures and clinical relevance. These findings support a stratified approach to IBD diagnosis and therapy, enabling more personalised disease management strategies.

## Plain language summary

Identification of the IBD subtype using machine learning

Inflammatory Bowel Disease (IBD) is a complex gastrointestinal disorder affecting millions worldwide. This groundbreaking study analyzed RNA sequencing data from 2490 adult patients, revealing three distinct molecular subtypes for both Ulcerative Colitis and Crohn’s Disease. By examining gene expression patterns in intestinal biopsies, researchers identified unique clusters characterized by different cellular processes like RNA processing, cytoskeletal dynamics, and stress responses. Each subtype showed specific gene upregulation and distinct molecular signatures. The research used Kmeans clustering and statistical analysis to link these subtypes with disease severity and regional variations. This innovative approach provides deeper insights into IBD’s molecular mechanisms, potentially paving the way for more personalized treatment strategies.

## Introduction

Inflammatory bowel disease (IBD), which includes Crohn’s disease (CD) and ulcerative colitis (UC), is a chronic inflammatory condition arising from the combined influence of genetic, immune and environmental factors, and primarily targeting the gastrointestinal tract (GIT).^1^ A careful balance needs to be maintained between immune tolerance and response during the dense microbial environment. When this equilibrium is disturbed, resulting in dysbiosis, can trigger intestinal inflammation, as observed in IBD.^2,3^ Although CD and UC exhibit similarities in clinical phenotypes, they differ substantially in anatomical involvement and pathological features. CD is typically marked by transmural inflammation that can affect any part of the GIT, particularly the terminal ileum, whereas UC is restricted to mucosal inflammation in the colon.^4^ Epidemiological data suggest that in the UK alone, UC affects approximately 397 per 100,000 people, and CD affects 276 per 100,000, with the global prevalence surpassing 6.8 million cases. This growing burden underscores the need for improved diagnostic precision and targeted care.^5–7^ IBD presents significant diagnostic and therapeutic challenges due to its complex aetiology and heterogeneous clinical manifestations.^8–10^ The non-specific nature of IBD symptoms often leads to misdiagnosis with other gastrointestinal conditions, such as IBS, colon cancer or infections, and current clinical methods fall short in capturing the molecular heterogeneity of the disease.^11,12^ Despite advances, precise subtyping remains elusive, limiting the potential for personalised treatment approaches. Transcriptomic technologies, particularly RNA sequencing (RNA-seq), have emerged as powerful tools to explore gene expression profiles associated with IBD. More importantly, this approach enables the discovery of distinct molecular subtypes by capturing disease-specific gene expression patterns, offering a pathway towards improved diagnosis and personalised therapy.^13–15^ RNA-seq studies have identified key differentially expressed genes (DEGs) involved in immune regulation, cell adhesion and inflammatory responses.^16–18^ Machine learning (ML) and artificial intelligence (AI) have further enhanced the utility of transcriptomic data, with ML models successfully predicting disease severity, outcomes and subtypes.^19–21^ While previous studies have applied clustering to identify UC subtypes using microarray and immune profiling,^22^ others have used RNA-seq or DNA methylation data to classify CD and UC or uncover potential biomarkers.^23,24^

Recent advances have applied ML to blood-based transcriptomic data for IBD classification. For example, a study developed a 17-gene qPCR assay predictive of disease prognosis,^25^ while another study identified FEZ1 as a non-invasive diagnostic biomarker using feature selection with random forest and LASSO models.^26^ Beyond transcriptomics, supervised ML using whole-exome sequencing (WES) data has shown potential in distinguishing CD from UC, using immune-related variant panels.^27^ Multi-omics approaches are also gaining attraction, with studies integrating genomics, transcriptomics, proteomics and metabolomics to improve IBD classification. One of the studies used such integration in the SPARC cohort to identify clinically relevant molecular subtypes,^28^ while others leveraged microbiome–metabolome data to develop robust diagnostic models.^29^ Additionally, post-surgical transcriptomic studies have identified gene signatures predictive of recurrence in CD and UC, including inflammatory and T-cell expression profiles.^30,31^

Despite these advancements, most prior studies are limited to one disease type, focus on a single omics layer or lack clinical validation. This study addresses these limitations by integrating transcriptomic data with clinical metadata and validating subtype discovery across both tissue and blood-derived samples. This study hypothesises that identifying subtypes of IBD beyond UC and CD could advance our understanding of IBD’s underlying mechanisms and pave the way for more personalised treatments, improving outcomes for patients. By applying unsupervised clustering and functional enrichment analysis to a large IBD cohort, this research aims to identify transcriptomic subtypes across both UC and CD and the key genes driving them, enabling a more comprehensive and clinically meaningful framework for IBD stratification.

## Methods

### Study design and datasets collection

This study utilises four RNA-sequenced gene expression datasets obtained from the Gene Expression Omnibus (GEO). The primary dataset consists of tissue biopsy count data from 2490 patients (GSE193677),^32^ while the validation dataset includes blood gene expression data from 1030 patients (GSE186507),^33^ both sourced from the same cohort. Two independent datasets were utilised for further validation: GSE137344^34,35^ and GSE235236.^36^ A flow diagram outlining the systematic search and selection of transcriptomic datasets is provided in Supplemental Figure 1. GSE193677 and GSE186507 datasets were procured from a study on molecular biomarkers in IBD conducted by Argmann et al.^37^ Biopsy samples from the primary dataset included patients with UC, CD and healthy controls (Table 1). It also provided a comprehensive view of IBD, incorporating diverse conditions and metadata attributes such as age, endoscopic severity and clinical measures, allowing for a detailed analysis of gene expression patterns across different disease profiles and stages. This study follows the STROBE (Strengthening the Reporting of Observational Studies in Epidemiology) reporting guidelines,^38^ and the completed STROBE checklist is provided in Supplemental Table 1.

**Table 1.** IBD datasets used in this study.

IBD dataset	Data type	Number of samples	Reference
(Primary or discovery)^32^	Bulk RNA-seq tissue biopsy counts data	CD = 1153 UC = 843 Control = 461	Argmann et al.^37^ (GSE193677)
(UC and CD validation)^33^	Blood transcriptome counts data	CD = 429 UC = 378 Control = 209	Argmann et al.^37^ (GSE186507)
(CD validation)^34^	Ileum transcriptome counts data	CD = 112 Control = 37	Mo et al.^22^ (GSE137344)
(UC validation)^36^	Bulk RNA-seq colon biopsy counts data	UC = 26 Control = 8	Garrido-Trigo et al., 2023^36^ (GSE235236)

CD, Crohn’s disease; IBD, inflammatory bowel disease; UC, ulcerative colitis.

### Data preprocessing

**Figure 1.**
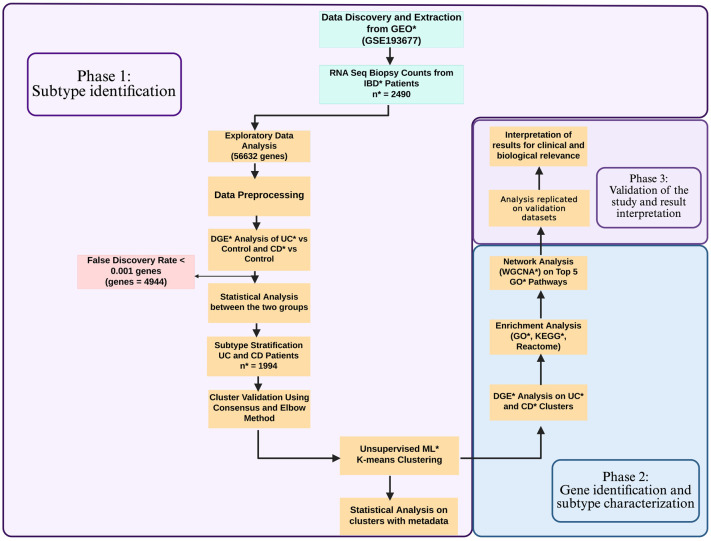
Workflow of data analysis displaying different steps and methodologies adapted in each phase. CD, Crohn’s disease; GEO, Gene Expression Omnibus; GO, gene ontology; IBD, inflammatory bowel disease; KEGG, Kyoto Encyclopaedia for Genes and Genomes; ML, machine learning; *n*, number of samples; UC, ulcerative colitis; WGCNA, weighted gene co-relation network analysis.

The entire data analysis and statistical computations were performed using the R programming environment.^39^ Raw counts data and metadata were filtered and cleaned to ensure consistency and alignment of datasets (Figure 1). Column names were standardised for uniformity. Samples with low counts, such as UC Pouch (*n* = 29) and CD Pouch (*n* = 4) from the primary dataset (GSE193677), and UC Pouch (*n* = 11) and CD Pouch (*n* = 3) from the GSE186507 validation dataset, were excluded to improve data quality and robustness of downstream analyses (Figure 1). From the GSE137344 validation dataset, UC (*n* = 44) and non-IBD (*n* = 1) samples were also excluded to focus on CD samples. Whereas CD (*n* = 22) samples were excluded from GSE235236 to focus on UC samples.

### Differential gene expression analysis

Differential gene expression (DGE) analysis was carried out on the filtered dataset, with control samples serving as the reference group (Supplemental Figure 2). The analysis considered UC, CD and controls as factors. Normalisation of raw count data was performed using the calcNormFactors function, followed by voom transformation^40^ to log-transform normalised counts. Subsequently, linear model fitting and empirical Bayes moderation were applied to identify significant genes.

DGE analysis was conducted using the Benjamini–Hochberg correction^40^ to minimise false positives and ensure comparability. A threshold of false discovery rate <0.001 was considered significant genes for UC and CD samples. Additional analyses utilised the cpm (counts per million) to log-transform expression values for downstream computations. To detect outlier samples and reduce dimensionality, principal component analysis (PCA) was performed on the scaled dataset. PCA plots of the first two principal components, representing the greatest variances, were analysed to identify and remove outlier samples. After preprocessing, the final dataset comprised 1153 CD samples with 2686 significant genes and 843 UC samples with 2258 significant genes.

### Subtype stratification using unsupervised ML

To divide the samples into clusters representing potential subtypes, clustering methods were evaluated to ensure minimal within-cluster differences. Hierarchical clustering, consensus clustering and *K*-means clustering all supported an equal number of clusters. However, *K*-means clustering was selected due to its computational efficiency in grouping data points based on proximity to cluster mean values, scalability to large datasets and interpretability in high-dimensional gene expression data. Compared to hierarchical or density-based clustering, *K*-means produced clearer, non-overlapping subgroup separations in preliminary tests and has been effectively applied in previous transcriptomic subtyping studies in IBD.^22^

To ensure the robustness of our clustering approach, we employed a multi-metric internal validation framework. First, we used the elbow method to determine the optimal number of clusters (*K* = 3), by plotting the within-cluster sum of squares (WCSS) against increasing values of *K*. To further validate this selection, we performed consensus clustering using 100 iterations of hierarchical clustering. Cluster stability was then assessed using the cumulative distribution function (CDF) and delta area plots, allowing us to evaluate how consistently samples grouped across iterations (Supplemental Figure 3).

We also assessed cluster robustness through bootstrap resampling (*n* = 100) and calculated the Jaccard similarity index for each cluster, which measures how consistently the same samples cluster together across resampling runs.^41^

To evaluate the quality of clustering in terms of internal structure, we calculated average silhouette widths.^42,43^ Finally, we applied the gap statistic to independently estimate the optimal number of clusters, by comparing observed WCSS to that expected under a null reference distribution (Supplemental Figure 4).

DGE analysis was subsequently performed on genes within each cluster using the non-transformed counts data and the limma-voom pipeline.^40^ Genes with adjusted *p*-values <0.001 and log2 fold change (log2FC) ⩾1 were considered significantly upregulated or downregulated in each cluster. Unlike the initial DGE analysis, which compared UC, CD and control groups to identify overall significant genes across the dataset, this refined DGE analysis specifically focused on differences within the identified clusters. This allowed for the identification of distinct gene expression profiles that define and differentiate each cluster, providing a more granular understanding of the heterogeneity within UC and CD subtypes.

### Clinical data analysis on UC and CD clusters

To understand the biological processes driving the clustering, metadata attributes (e.g. age, region, gender, endoscopic severity and clinician’s measures) were incorporated into the clusters. A Chi-squared test^39^ was employed to determine significant associations between categorical metadata variables (such as gender) and the identified clusters.

For continuous variables like age, an analysis of variance (ANOVA) test^39^ was performed. This allowed identification of statistically significant differences in the means of continuous variables across UC and CD clusters, enhancing the understanding of metadata variation across subgroups.

### Selection of genes and gene set enrichment analysis

Gene selection was based on the results of DGE analysis within each *k*-means cluster. Genes with adjusted *p*-values <0.001 and log2FC ⩾1 were identified as significantly upregulated or downregulated in each cluster. These significant genes were then subjected to gene set enrichment analysis (GSEA) to determine their functional relevance and contribution to the clustering. GSEA was performed using resources such as the Gene Ontology Consortium (GO), Kyoto Encyclopaedia of Genes and Genomes (KEGG) and Reactome^44^ databases to identify key genes and pathways responsible for the subgrouping of samples. GO analysis provided insights into biological processes, molecular functions and cellular components, while KEGG focused on metabolic pathways and molecular interaction networks. Reactome characterised immune and disease-related pathways. Top upregulated and downregulated pathways were combined to identify the most relevant pathways for each UC and CD cluster. Top enriched pathways were selected based on their enrichment scores from the GSEA, and genes responsible for those pathways were extracted for module analysis using weighted gene co-expression network analysis (WGCNA).

### Weighted gene co-expression network analysis

WGCNA^45^ is a useful tool for determining the modules (clusters) in high-dimensional datasets. These modules can later be used as a dimensionally reduced set of clusters to study the relationships between modules that are co-expressed or for feature selection studies. The counts data of genes extracted from the top pathways of UC and CD clusters were passed on to the picksoftthreshold^45^ function of WGCNA, the resulting scale independence and mean connectivity were plotted to find the optimal value of power for module formation. A value that corresponds to above 0.85 in scale independence and below 100 in mean connectivity was selected. With a minimum module size of 30, the cutreeDynamic^45^ function was used to identify the modules, and the moduleEigengenes^45^ function was used to calculate the eigengenes. Similar modules found were merged to reduce redundancy.

It is worth noting that ‘clusters’ in this study refer to patient subgroups identified through *K*-means clustering based on transcriptomic profiles, whereas ‘modules’ represent a set of co-expressed genes derived from WGCNA. While clusters described the patterns across patient samples, modules were created to explore gene–gene relationships and underlying biological pathways within those patient groups.

### Validation of the subtype’s identification

To ensure the reliability and reproducibility of the findings, the entire analysis was replicated on the validation dataset (GSE186507), which consisted of blood gene expression data from 1030 patients. While the primary dataset (GSE193677) contained tissue biopsy data, the validation dataset provided an independent, biologically distinct sample type, enabling the assessment of the model’s robustness across different specimen types. We calculated average silhouette widths and applied the gap statistic to independently estimate the optimal number of clusters, by comparing observed WCSS to that expected under a null reference distribution (Supplemental Figure 5).

To further strengthen the results, an additional external RNA-seq validation dataset, GSE137344, was incorporated for CD. This dataset consisted of tissue biopsy samples, aligning more closely with the primary dataset in terms of biological material and enabling cross-cohort consistency checks. The entire analytical workflow, including data preprocessing, normalisation, clustering, internal validation (Jaccard index, silhouette score, gap statistic) and downstream differential expression and enrichment analysis, was independently replicated for both external datasets.

The same statistical cut-offs were applied on GSE186507 validation dataset to maintain consistency. However, to avoid overly stringent filtering in the GSE137344 dataset, given its relatively low gene count after preprocessing, a relaxed threshold of adjusted *p*-value <0.05 was used prior to clustering analysis. DGE analysis used the thresholds of adjusted *p*-value <0.001 and log2FC ⩾1 to identify significant genes. Similarly, clustering and pathway enrichment analysis followed the same *k*-means and GSEA methodologies as the primary dataset.

A detailed list of all tools and R packages used in this study, along with their respective versions and purposes, is provided in Supplemental Table 2 to ensure project documentation, transparency and reproducibility.

## Results

### *K*-means clustering identified three subtypes

**Figure 2.**
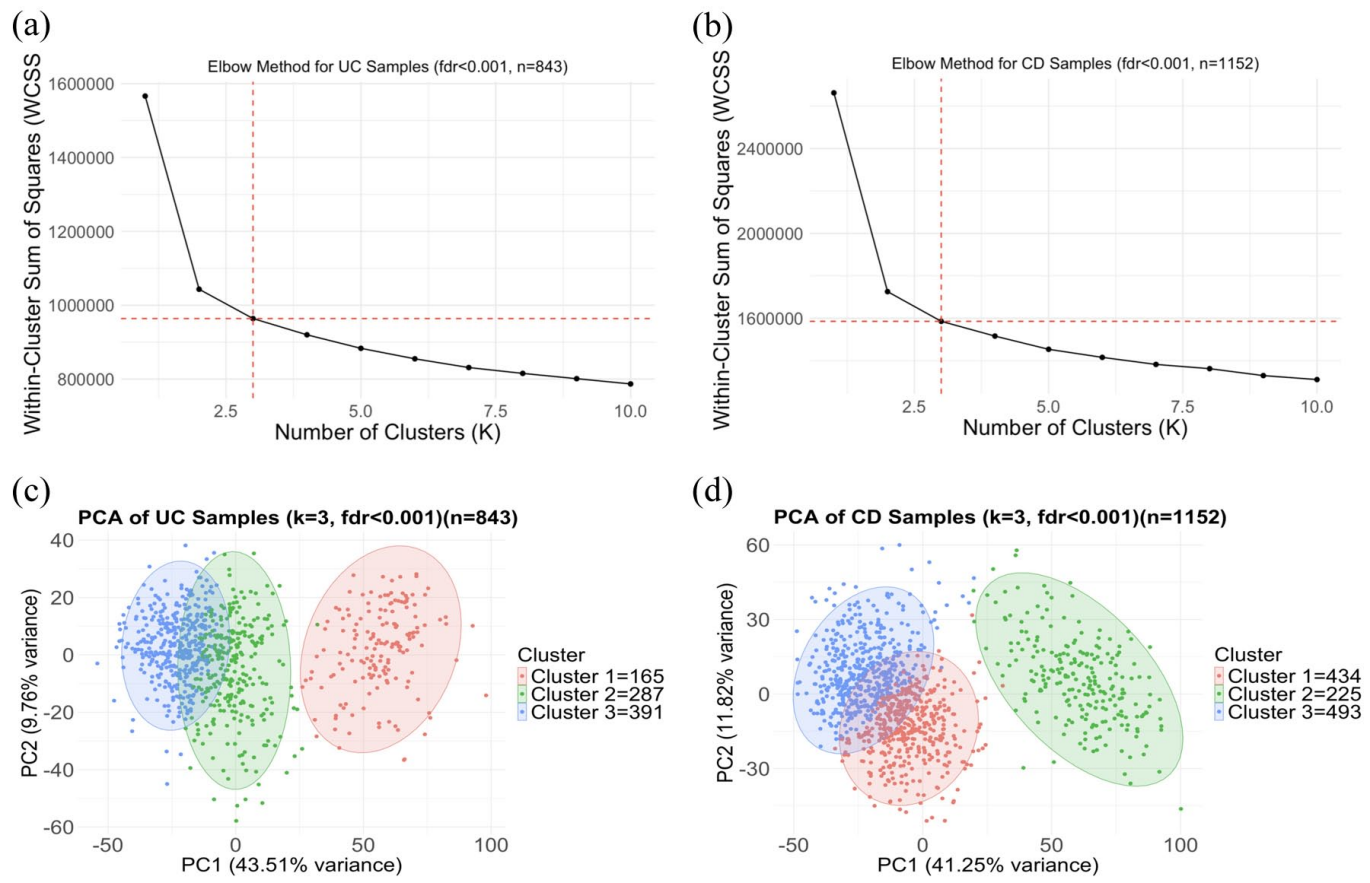
Results from *K* value selection and clustering analysis. (a, b) Elbow plot showing *k* = 3 as the appropriate *K*-value for UC and CD datasets, respectively. (c, d) PCA plot of *K*-means clustering showing three clusters within UC and CD samples, respectively. (a, b) *X*-axis represents the number of clusters (*k*), *Y*-axis depicts WCSS and the red dotted line indicates the optimum value of *k*. (c, d) *X*-axis and *Y*-axis show PC1 and PC2, respectively, with variance in %. CD, Crohn’s disease; PCA, principal component analysis; UC, ulcerative colitis; WCSS, within-cluster sum of squares.

Using the WCSS plot, *K* = 3 was determined to be the optimal number of clusters, providing the best balance between data compactness and interpretability. *K*-means clustering analysis resulted in three distinct subtypes for both the UC and CD datasets. For UC, the clusters comprised 165, 287 and 391 samples, while for CD, the clusters included 434, 225 and 493 samples, respectively (Figure 2).

To assess the reproducibility and quality of these clusters, we applied a multi-metric internal validation strategy. Consensus clustering based on 100 iterations of hierarchical clustering confirmed *K* = 3 as the most stable solution, as indicated by stabilisation in the CDF and delta area plots. Additionally, the gap statistic independently supported *K* = 3 by maximising the gap between observed and expected WCSS.

Bootstrap resampling (*n* = 100) further evaluated the stability of each cluster using the Jaccard similarity index. *K*-means clustering showed high reproducibility across both conditions. For CD, Cluster 1 demonstrated the strongest stability (Jaccard = 0.752), while Clusters 2 and 3 remained moderately stable (0.62–0.67). By contrast, hierarchical clustering produced less consistent results, particularly for Cluster 3 (Jaccard = 0.435). Average silhouette scores, which assess how well-separated each sample is from neighbouring clusters, were relatively low across methods (<0.2), as is common in transcriptomic data. However, *K*-means achieved the highest average silhouette width (0.092), with Cluster 2 showing modest separation (0.11), supporting the presence of meaningful subgroup structure (Table 2).

**Table 2.** Number of samples and significant genes (FDR < 0.001) in each UC and CD cluster upon DGE analysis.

Cluster comparison	Number of samples (UC cluster)	Number of samples (CD cluster)	Significant genes (UC)	Significant genes (CD)
Cluster 1 vs others	165	434	2000	2218
Cluster 2 vs others	287	225	1744	2416
Cluster 3 vs others	391	493	1972	2497

CD, Crohn’s disease; DGE, differential gene expression; FDR, false discovery rate; IBD, inflammatory bowel disease; UC, ulcerative colitis.

The clustering revealed clear segregation of samples, suggesting distinct transcriptomic profiles for each subtype. These clusters were subjected to further analysis to identify their unique gene expression patterns, pathways and potential clinical implications. This classification lays the groundwork for a more refined understanding of UC and CD heterogeneity.

### DGE analysis

**Figure 3.**
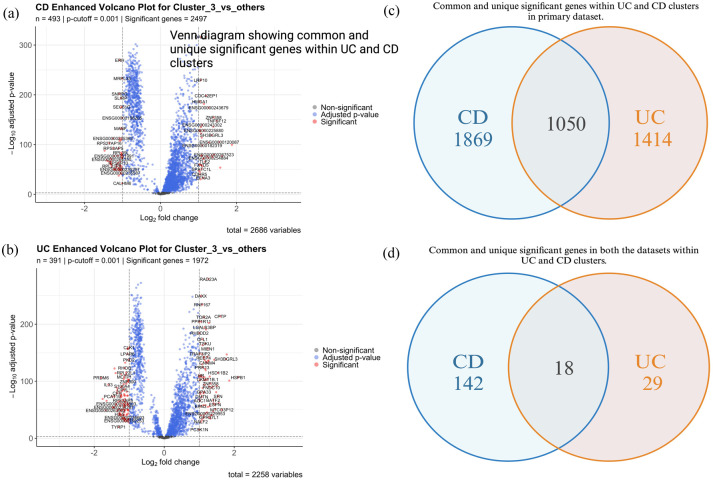
Differential gene expression analysis on UC and CD samples with adjusted *p*-value < 0.001 and Log2FC = 1. (a, b) Enhanced volcano plot of CD cluster 3 and UC cluster 3 showing statistically significant genes (c). Venn diagram showing common and unique significant genes in UC and CD samples across the clusters in the primary dataset (d). Venn diagram showing common and unique significant genes in both the primary and validation dataset across all UC and CD clusters. CD, Crohn’s disease; log2FC, log 2 fold change; UC, ulcerative colitis.

DGE analysis (Figure 3) between UC, CD and control samples identified 2258 significant genes in UC versus Control and 2686 in CD versus Control, which were used as the basis for subtype identification. These genes provided insights into the overall transcriptomic differences between diseased and healthy states.

Subsequent DGE analysis of the three *k*-means clusters within UC and CD datasets refined these findings by identifying genes that were uniquely upregulated or downregulated in each cluster (Table 2). This cluster-specific analysis revealed distinct transcriptomic profiles for the subtypes, highlighting genes that potentially drive the unique characteristics of each group. Interestingly, 1050 significant DEGs were found to be shared between UC and CD clusters (Figure 3).

### Linking clinical data with subtypes

Statistical analyses were performed to evaluate the association between clinical metadata and the identified clusters. The Chi-squared test showed no significant association between clusters and ‘IBD Clinician Measure’ (Active, Inactive, NA) in both UC (*p* = 0.076) and CD (*p* = 0.074). Similarly, ‘Gender’ showed no significant association in UC (*p* = 0.2593) and CD (*p* = 0.2919).

Conversely, ‘IBD Endo Severity’ (Severe, Mild, Moderate, Inactive) demonstrated a significant association with clusters in both UC (*p* = 0.000263) and CD (*p* = 0.007006). Specifically, Cluster 3 in both UC and CD exhibited a higher proportion of samples classified as moderate to severe, whereas Cluster 1 was predominantly composed of inactive or mild cases. These associations are illustrated in Supplemental Figure 6.

Additionally, ‘IBD Region’ (left colon, right colon, rectum, sigmoid, transverse, ileum) exhibited a strong association with clusters in both UC and CD datasets (*p* < 0.000001). The ANOVA test revealed significant differences in ‘Age’ across UC clusters (*p* = 0.0000345), although no significant differences were observed in CD clusters (*p* = 0.285). The detailed results from statistical data analysis for linking clinical data attributes with subtypes identified are provided in Supplemental Table 3.

### Pathway and GSEA

**Figure 4.**
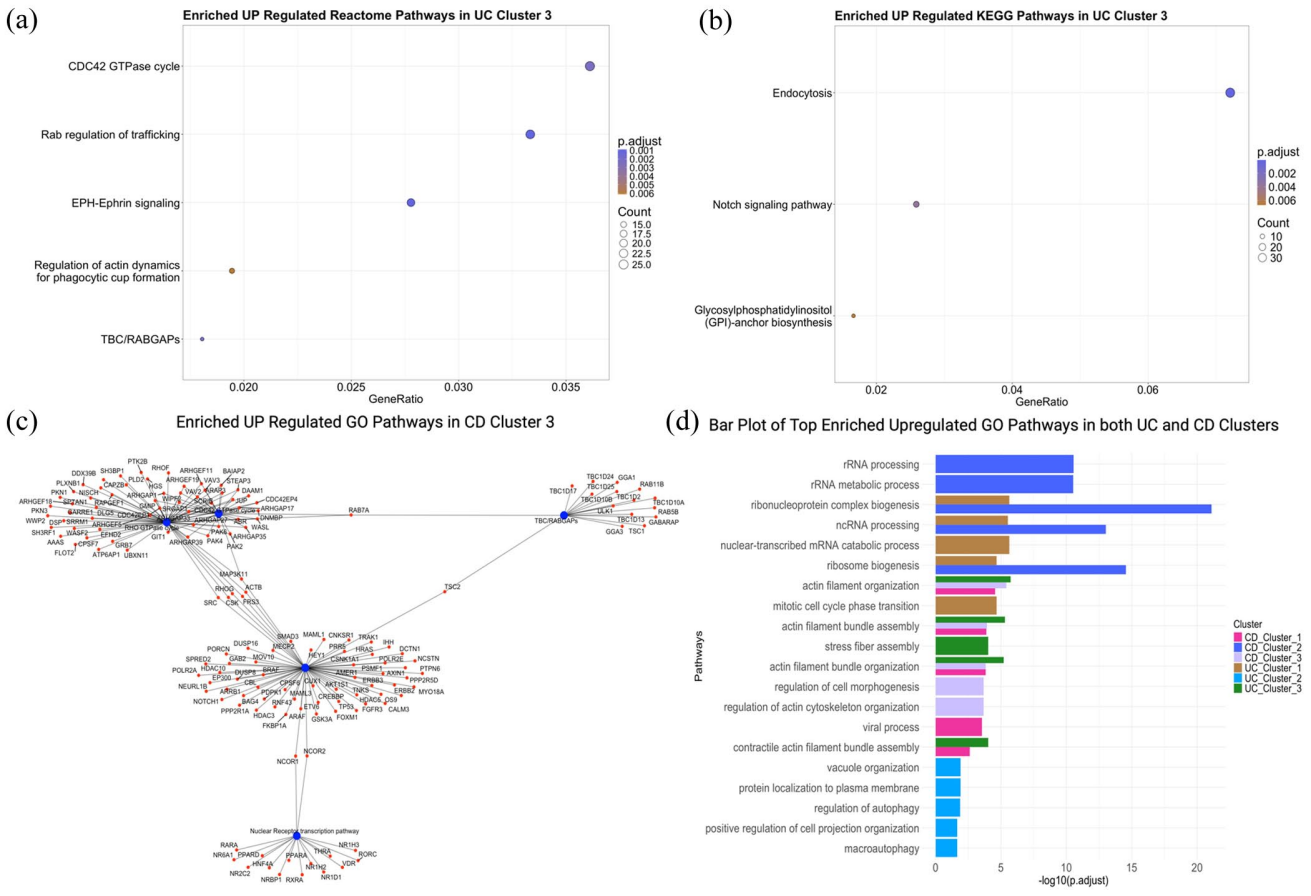
Visualisation of enriched pathways in UC and CD clusters. (a, b) Dot plot showing the top 5 enriched upregulated Reactome and KEGG pathways, respectively, in UC Cluster 3. Each bubble represents a pathway, with its size indicating the number of genes involved. (c) Network plot of the top 5 enriched upregulated GO pathways in CD Cluster 3. Red nodes represent pathways, while blue nodes represent genes. Edges (lines) denote interactions or associations between pathways and genes. (d) Combined bar plot illustrating the top enriched upregulated GO pathways across UC and CD clusters. CD, Crohn’s disease; GO, gene ontology; KEGG, Kyoto Encyclopaedia of Genes and Genomes; log2FC, log 2 fold change; UC, ulcerative colitis.

Pathway enrichment analysis identified key biological processes enriched in both UC and CD clusters, including translation, proteasome activity and RNA processing, which were upregulated across clusters (Figure 4). By contrast, pathways related to protein processing, degradation and chromatin modifications were consistently downregulated, suggesting their critical involvement in disease progression.

Furthermore, this study identified potential therapeutic targets based on the affected pathways and systems. These findings provide a foundation for further exploration of cluster-specific interventions in IBD treatment.

### Weighted gene co-expression network analysis

**Figure 5.**
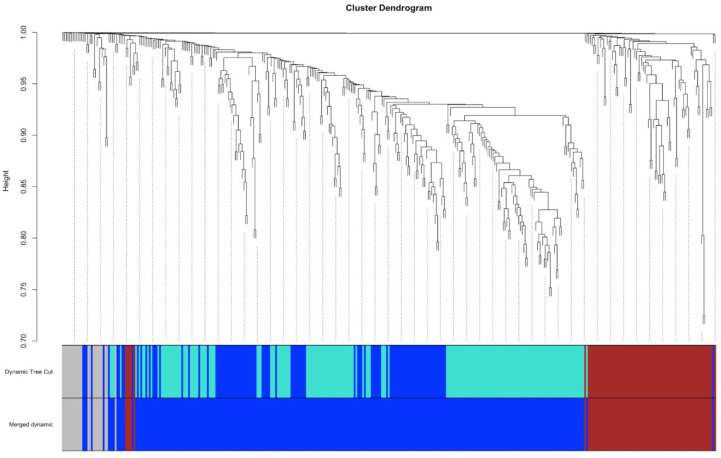
A cluster dendrogram illustrating the arrangement of clusters of genes produced by WGCNA. On the bottom, the ‘Dynamic Tree Cut’ and ‘Merged Dynamics’ show the module colour assigned to the branch. WGCNA, weighted gene co-expression network analysis.

Weighted gene co-expression network analysis was performed on the genes identified from the top pathways found in the GSEA. This analysis revealed three distinct modules for both UC and CD datasets, represented by different colours (Figure 5). These modules consisted of highly correlated genes, highlighting the structured relationships among genes within the top enriched pathways.

The identification of three modules aligns with the clustering results, further validating the presence of distinct subgroups within UC and CD. While the specific biological processes associated with each module were not analysed, the results provide a robust framework for understanding the organisation of gene expression patterns within IBD subtypes. The modular approach emphasises the interconnectedness of key genes and pathways and sets the stage for future investigations into the functional implications of these modules.

### Characterisation of clusters from GSEA

#### Crohn’s disease

**Figure 6.**
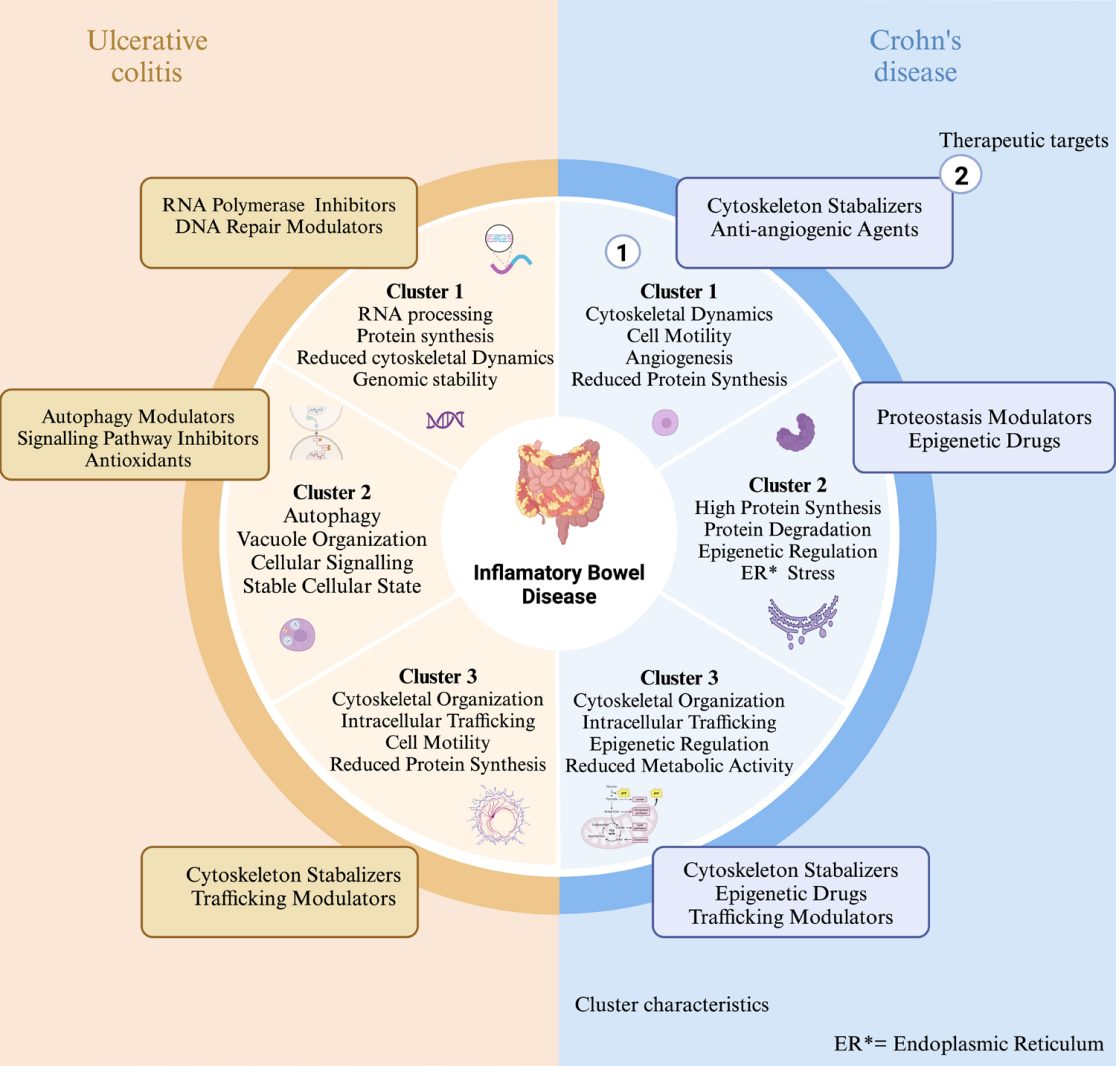
Characterisation of clusters in the primary dataset shows (1) the characteristics of the cluster and (2) the potential therapeutic targets for that cluster.

Cluster 1 consisting of 434 samples was characterised by upregulation of cytoskeletal dynamics, cellular motility and a unique response to viral infections. Protein synthesis pathways were notably downregulated, suggesting reduced metabolic activity in this cluster. Potential therapeutic targets include cytoskeleton stabilisers and anti-angiogenic agents. Cluster 2, consisting of 225 samples, demonstrated a strong focus on protein synthesis, folding and degradation, indicating a state of high metabolic stress and activity. This cluster may reflect a rapidly progressing form of CD.^46^ Cytoskeletal and signalling pathways were downregulated, highlighting less emphasis on maintaining cell shape and signalling processes. Proteostasis modulators and epigenetic drugs were identified as potential therapeutic targets for this subtype. Cluster 3, consisting of 493 samples, exhibited significant upregulation in cytoskeletal organisation, intracellular trafficking and epigenetic regulation, while showing reduced activity in protein synthesis pathways. This suggests a prioritisation of cellular structure and signalling maintenance over metabolic activity. Therapeutic targets for this cluster include cytoskeleton stabilisers, epigenetic drugs and trafficking modulators (Figure 6). Across all CD clusters, commonly expressed genes included *COX1, CDH1* and *SF3B1*, which are involved in mitochondrial activity, cell adhesion and RNA splicing, respectively. The selection of these genes was also supported by previous studies that linked these genes to IBD-related processes.

#### Ulcerative colitis

Cluster 1, consisting of 165 samples, was distinguished by high RNA processing and protein synthesis activity, along with chromatin remodelling and DNA repair mechanisms. This may reflect a need for genomic stability and rapid cell turnover. Cytoskeletal dynamics and intracellular trafficking were deprioritised in this cluster. RNA polymerase inhibitors and DNA repair modulators were identified as potential therapeutic targets. Cluster 2, consisting of 287 samples, showed upregulation of autophagy, vacuole organisation and cellular signalling pathways, suggesting a focus on maintaining cellular homeostasis and responding to metabolic stress. This subtype appears to manage cellular damage and structural integrity. Autophagy modulators, signalling pathway inhibitors and antioxidants were identified as potential therapeutic options. Cluster 3, consisting of 391 samples, emphasised cytoskeletal organisation and intracellular trafficking, with processes supporting cell shape, motility and structural integrity. This cluster prioritised stability in cellular structure and signalling over metabolic activity, suggesting a more stable cellular state. Therapeutic targets for this cluster include cytoskeleton stabilisers and trafficking modulators (Figure 6). All UC clusters shared the expression of *COX1, TMSB10* and *ACTB* genes involved in mitochondrial function, cytoskeletal organisation and inflammatory responses. The selection of these genes was also supported by previous studies that linked these genes to IBD-related processes.

UC clusters upon visualisation using heatmap revealed similar subgrouping within the UC samples, that is, there are three distinct clusters within the UC dataset.

**Figure 7.**
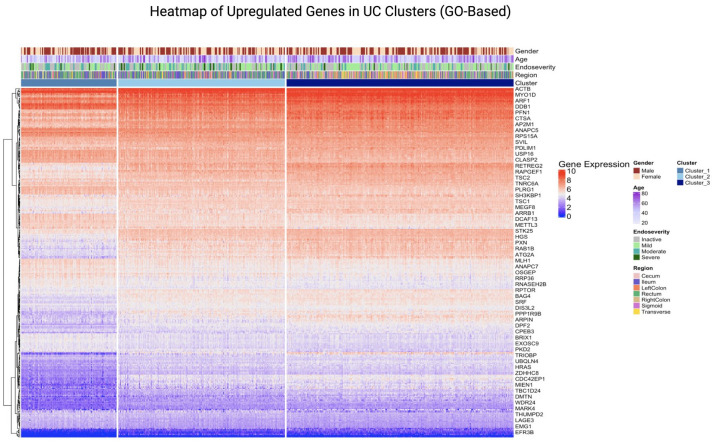
Heatmap of genes from the top 5 upregulated GO pathways in UC (primary dataset). This heatmap visualises the expression levels of genes from the top 5 upregulated GO pathways in UC clusters within the primary dataset. Samples are annotated by clusters and clinical features, including gender, age, endoscopic severity and region. The plot highlights the differential expression patterns of genes across clusters, emphasising their roles in upregulated GO pathways. GO, gene ontology; UC, ulcerative colitis.

**Figure 8.**
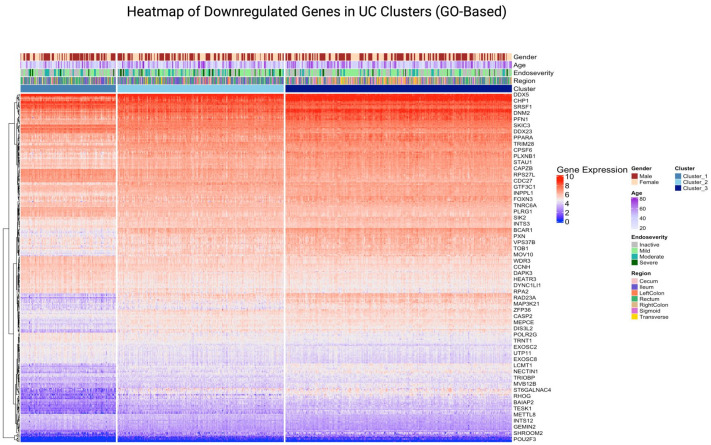
Heatmap of genes from the top 5 downregulated GO pathways in UC (primary dataset). This heatmap visualises the expression levels of genes from the top 5 downregulated GO pathways in UC clusters within the primary dataset. Samples are annotated by clusters and clinical features, including gender, age, endoscopic severity and region. The plot highlights the differential expression patterns of genes across clusters, emphasising their roles in downregulated GO pathways. GO, gene ontology; UC, ulcerative colitis.

On creating a heatmap of the genes extracted from the top 5 pathways, we can see the clustering of genes as well as the genes that are highly expressed. The higher and lower expressed regions (Figures 7 and 8) could indicate that these genes and their associated pathways are probably responsible for the progression of the disease. The part of the heatmap that shows distinct patterns of gene expression suggests that each cluster had a differentiating gene expression level, indicating that there are three potential subgroups within the UC dataset. Network analysis further resulted in modules (clusters) of highly correlated genes, named after colours.

## Validation

We performed validation of the subtypes on two independent tissue types: blood samples and biopsy samples.

### Validation on blood samples

#### UC subtypes

The validation dataset confirmed the presence of stable clusters in UC when *K* was set to 3, consistent with the discovery dataset (Supplemental Figures 7 and 8). DGE analysis identified 491 significant genes for UC in the validation dataset.

Cluster distribution for UC showed that Cluster 1 consisted of 103 samples, Cluster 2 had 188 samples and Cluster 3 contained 87 samples, demonstrating distinct grouping within the validation dataset.

A comparison between the discovery and validation datasets revealed 29 genes common across UC clusters, including *ARPIN, ERBB2, ZBTB7B* (*ThPOK*) and *NLRX1*. These genes are implicated in epithelial proliferation, immune modulation and inflammatory responses, validating their importance in UC subtyping.

Clinical variables further supported the clustering results in UC. IBD Endo Severity was significantly associated with UC clusters, reflecting its role in defining disease activity. The IBD Clinician Measure also showed significant associations with UC clusters, suggesting its relevance in assessing disease subtypes. Gender distribution was significantly different across UC clusters, indicating potential gender-specific influences in UC subtypes. Additionally, age displayed significant differences across clusters, highlighting its impact on disease heterogeneity and progression in UC (Supplemental Figure 9).

Furthermore, pathway analysis of UC and CD clusters showed consistent enrichment of upregulated GO pathways (Supplemental Figure 10). Gene expression heatmaps of top upregulated and downregulated GO pathways in UC validation samples illustrated distinct expression signatures and clustering behaviour (Supplemental Figures 11 and 12).

#### CD subtypes

For CD, the validation dataset similarly confirmed the presence of stable clusters when *K* was set to 3 (Supplemental Figures 7 and 8). DGE analysis identified 1857 significant genes for CD in the validation dataset.

Cluster distribution for CD showed that Cluster 1 consisted of 88 samples, Cluster 2 had 190 samples and Cluster 3 contained 150 samples, further validating the distinct subgrouping within the dataset.

A comparison between the discovery and validation datasets showed 142 genes common across CD clusters, including *IL17RA, NLRX1, ERBB2, CFH* (Complement Factor H), *ZBTB7B, RXRA, GADD45G* and *ARPIN.* These genes are associated with *Th17* signalling, immune dysregulation, stress response and inflammatory pathways, reinforcing their roles in defining CD subtypes.

Clinical variables also demonstrated significant clustering associations in CD. IBD Endo Severity was significantly associated with CD clusters, underscoring its relevance in defining disease activity. However, the IBD Clinician Measure did not show significant associations in CD clusters, suggesting differences in the utility of clinician-based assessments between UC and CD. Age showed significant differences across CD clusters, reflecting its influence on disease progression and heterogeneity. Unlike UC, gender distribution was not significantly associated with CD clusters (Supplemental Figure 9).

### Validation on biopsy samples

#### CD subtypes

We used GSE137344 for cluster distribution validation for CD patients. Results showed that Cluster 1 consisted of 43 samples, Cluster 2 had 40 samples and Cluster 3 contained 29 samples. DGE analysis identified 958, 2044 and 1147 significant genes in clusters 1, 2 and 3, respectively (Supplemental Table 4). This external validation dataset confirmed the presence of stable clusters when *K* was set to 3 (Supplemental Figures 13 and 14).

Cluster 1 showed strong upregulation of adaptive immune processes, including lymphocyte differentiation, antigen receptor signalling and B-cell activation, alongside consistent downregulation of oxidative phosphorylation and aerobic respiration, indicating a highly immune-reactive and metabolically reprogrammed phenotype. Cluster 2 was enriched in pathways related to lipid and fatty acid metabolism, biological oxidation and peroxisomal function, while immune regulatory pathways such as cytokine signalling and B cell receptor signalling were significantly downregulated, suggesting a metabolically altered but less inflamed subtype. Cluster 3 was characterised by elevated expression of cytokine production, leukocyte-mediated immunity and cell cycle progression, with prominent activation of interleukin signalling, DNA replication and ER protein processing, coupled with suppression of catabolic and oxidative processes. Together, these findings reveal immune and metabolic divergence across CD subtypes, with consistent involvement of lipid metabolism, cytokine signalling and mitochondrial function across clusters.

Clinical and demographic variables were evaluated to assess their association with CD clusters derived from transcriptomic profiling. Age showed a non-significant trend across clusters, suggesting mild heterogeneity without strong influence on cluster structure. Gender distribution did not significantly differ across clusters, nor did ancestry (Supplemental Figure 15). These findings support the biological relevance of the CD clustering and reduce concerns of potential confounding due to patient metadata.

A comparison between the discovery and validation datasets showed a substantial overlap of 244 genes across all the clusters, including *SMAD7, GSK3B, MAP3K5, RORC, FUT3, EPS8, IL22RA1, HDAC10, RXRA* and *SLC22A5* across all clusters. These genes are associated with critical immune regulation, epithelial barrier integrity, Wnt signalling, apoptosis, T-cell differentiation and lipid metabolism pathways, reinforcing their roles in defining CD molecular subtypes. Several of these genes (e.g. *SMAD7, GSK3B, RORC* and *IL22RA1*) have previously been reported as relevant in IBD pathogenesis and therapeutic response, suggesting biological consistency between datasets.^47,48^ These genes also included genes such as *ARPIN, ERBB2* and *ZBTB7B*, which also appeared in the comparison between the discovery and blood validation datasets. These genes are associated with immune regulation, epithelial integrity and mucosal repair pathways, reinforcing their roles in defining CD subtypes (Supplemental Figure 16).

Gene expression heatmaps of top upregulated and downregulated GO pathways in GSE137344 CD validation samples show distinct expression signatures and clustering behaviour (Supplemental Figures 17 and 18).

#### UC subtypes

We also performed exploratory clustering analysis on GSE235236 as independent validation cohorts to assess the reproducibility of transcriptional UC subtypes identified in our discovery dataset. This dataset was selected based on inclusion criteria such as RNA-seq data from colonic tissue biopsies of treatment-naïve adult UC patients, closely mirroring the clinical context of our primary cohort.

The overall PCA using Hierarchical, *k*-means and consensus clustering showed separation between three UC clusters (Supplemental Figure 19).

## Discussion

### UC and CD subtypes in the discovery cohort

IBD is a chronic condition usually characterised by two main types of diseases, UC and CD, both of which affect the GIT. Due to its inflammatory nature, there are systemic changes that can be observed even at the transcriptomic level.^49^ The diagnosis of IBD relies heavily on endoscopic procedures, which are usually not detected at early stages. A study conducted by Mo et al.^22^ identified three distinct, stable UC subtypes through consensus analysis on microarray data, characterised by differences in immune activation, metabolic regulation and epithelial cell function. Our study not only replicates this three-subtype structure in UC using large-scale RNA-seq and gene expression profiles, but also extends it to CD, uncovering three transcriptomic clusters with distinct molecular and clinical characteristics. Notably, the UC subtypes identified in our study showed comparable patterns, one enriched for immune and autophagy pathways, another for RNA processing and cell turnover and a third dominated by cytoskeletal and structural pathways, mirroring the functional divergence reported by Mo et al.^22^ While similar biological patterns were observed, further comparative studies are needed to definitively confirm the overlap between the clusters identified in the two studies. Unlike prior studies limited to UC or small cohorts,^22^ we integrated clinical metadata, such as endoscopic severity and anatomical region, with cross-tissue validation across intestinal biopsies and blood samples. This comprehensive framework provides more robust, reproducible and clinically meaningful IBD subtyping.

Recent advancements in ML and multi-omics have significantly enhanced our understanding of IBD heterogeneity. Biasci et al.^25^ developed a 17-gene qPCR-based blood assay capable of predicting disease prognosis in newly diagnosed IBD patients, marking a step towards personalised therapy. Similarly, Delkhah^26^ identified FEZ1 as a potential non-invasive blood biomarker for IBD through transcriptomic analyses, highlighting the role of autophagy in disease pathogenesis. In the realm of genomics, Stafford et al.^27^ utilised WES data combined with supervised ML algorithms to distinguish between CD and UC, demonstrating the potential of genomic data in disease classification. Beyond single-omics approaches, Preto et al.^28^ integrated genomics, transcriptomics and proteomics data from the SPARC IBD cohort, successfully identifying novel biomarkers and patient subgroups, thereby underscoring the utility of multi-omics integration in uncovering disease mechanisms. Complementing these findings, Ning et al.^29^ conducted a cross-cohort integrative analysis of microbiome and metabolome data, revealing consistent microbial and metabolic signatures associated with IBD, which could serve as robust diagnostic markers. Additionally, studies by Ngollo et al.^30^ and Gonsky et al.^31^ have identified gene expression profiles in surgical ileal mucosa and blood T cells, respectively, that are predictive of postoperative recurrence in CD, emphasising the prognostic value of transcriptomic analyses in clinical settings. Collectively, these studies highlight the transformative impact of integrating ML with diverse omics data in refining IBD classification and prognostication. Our study builds upon these findings by employing unsupervised ML on RNA sequencing data from both UC and CD patients, identifying three distinct transcriptomic subtypes in each disease. Notably, these subtypes correlate with clinical parameters such as endoscopic severity and anatomical disease location, providing a more comprehensive framework for IBD stratification. The integration of clinical metadata with transcriptomic data enhances the reproducibility and translational potential of our findings.

### Mechanistic aspects of the genes in UC and CD clusters

In this study, we performed GSEA on DEGs identified from UC and CD and found that *ARPIN, NLRX1* and *ErbB2* consistently appear across both datasets (primary and validation) in UC clusters, suggesting a role in epithelial cell proliferation and inflammation as markers for UC subtypes.^50,51^ All the UC clusters of the primary dataset had *COX1, TMSB10* and *ACTB* genes in them, indicating involvement in mitochondrial function, cytoskeletal organisation and inflammatory responses.^52–54^ In case of CD, *IL17RA, CFH, RXRA* and *GADD45G* consistently appear across both datasets (primary and validation), suggesting a role in Th17 cell signalling, immune dysregulation, inflammation and stress response as markers for CD subtypes.^55–58^ All CD clusters of the primary dataset had *COX1, CDH1* and *SF3B1* genes as commonly expressed genes. These genes are related to mitochondrial activity, cell adhesion and RNA splicing. Indeed, these genes had been proven to be significant with various components of IBD, which is consistent with our study.^52,59,60^ We speculate that these genes could be disease-defining genes. GSEA also helped us to characterise each subtype of IBD, all of which had previous studies to link them to IBD. Looking within each disease type, we found that Autophagy regulation, RNA modification and genomic stability and cytoskeletal organisation were the main factors for the subtyping of UC.^61–64^ In the case of CD, we found that cytoskeletal dynamics, ER stress and protein synthesis and epigenetic regulation were the key factors responsible for subtyping.^65–67^ Moreover, *COX1, COX3, CDH1* and *ERBB3* were found highly expressed in all the UC and CD clusters possess significant risk of cancer and, *SF3B1* gene associated with hematologic malignancy, *APLP2* linked to neurodegenerative disease like Alzheimer’s, *MYH9* gene linked to kidney and haematological disorders and *TABPP* gene involved in autoimmune diseases were found to be common in all UC and CD clusters.^68–70^ While all these characteristics of the clusters that were found in this analysis have links to previous studies that validate their presence in the results, there are still a lot of unknowns in the case of IBD. In a study conducted by Tang et al.,^71^ a similar analysis was performed, and clustering was done on UC genes with the major difference being that the analysis was done on DEGs that are cuproptosis-associated. They found two subtypes of UC through consensus clustering that showed significant difference in gene expression patterns.^71^ These promising results further enhance our understanding of the underlying pathogenesis of IBD.

Interestingly, the study by Mian et al.^72^ also identified three clusters (*k* = 3) using unsupervised ML, but with a focus on routinely collected electronic health records (EHR). Their clustering approach highlighted key features such as faecal calprotectin levels, BMI, marital status and duration of advanced therapy. The convergence on three clusters in both transcriptomic and EHR-based analyses underscores the robustness of this stratification and suggests a potential universal pattern of subgrouping among IBD patients.

### Linking clinical features and clusters

In this study, each identified cluster in both UC and CD was evaluated against various clinical and demographic parameters. While no significant association emerged between clusters and the ‘IBD Clinician Measure’ in UC (*p* = 0.076) and CD (*p* = 0.074), nor between clusters and ‘Gender’ in UC (*p* = 0.2593) and CD (*p* = 0.2919), strong associations did appear for other clinical variables. In particular, ‘IBD Endo Severity’ (Severe, Mild, Moderate, Inactive) was significantly associated with the clusters in both UC (*p* = 0.000263) and CD (*p* = 0.007006), suggesting that the transcriptomic signatures characterising these clusters may reflect or correlate with mucosal disease severity as seen endoscopically. Similarly, ‘IBD Region’ (left colon, right colon, rectum, sigmoid, transverse, ileum) showed highly significant associations in UC and CD (*p* < 0.000001), indicating that anatomical location of involvement may be another important component of the molecular heterogeneity underlying IBD. Interestingly, while ‘Age’ was significantly different across UC clusters (*p* = 0.0000345), no such association was observed in CD (*p* = 0.285), potentially hinting at age-related transcriptional changes that manifest more prominently in UC than in CD.

When combined with the GSEA and network analyses, these clinical correlations help contextualise the molecular differences captured by unsupervised clustering. For example, UC clusters emphasising autophagy, RNA modification and cytoskeletal organisation may align more closely with severe endoscopic changes in certain segments of the colon than clusters where these pathways are downregulated. Similarly, in CD, the identified subtypes with distinct cytoskeletal dynamics, protein synthesis or epigenetic regulation appear to dovetail with the degree of endoscopic inflammation and the anatomic regions most affected.

### Validation and robustness of clustering

The validation dataset confirmed the stability and reproducibility of the clustering results, with three distinct subtypes identified for both UC and CD. Molecular overlaps between the primary and validation datasets, such as the consistent identification of key genes like *ARPIN, ERBB2, NLRX1* and *ZBTB7B*, reinforce the robustness of the clustering. These genes, linked to epithelial proliferation, immune modulation and inflammatory responses, emerged as significant markers for UC subtypes. Similarly, shared genes in CD clusters, such as *IL17RA, CFH* and *RXRA*, highlight conserved molecular pathways, including immune dysregulation and stress responses.

The identification of 18 shared genes between UC and CD, including *CMC1, NLRX1* and *CFH*, suggests common molecular mechanisms underlying both conditions. These genes point to pathways related to mitochondrial function, cytoskeletal organisation and cellular stress responses, providing potential targets for universal IBD therapies.

Clinical associations further supported the clustering. Strong correlations between IBD Endo Severity and clusters in both UC and CD linked molecular subtypes to disease activity. Significant age-related differences in UC clusters and gender-specific influences further underscored the demographic and biological heterogeneity of the disease.

The external CD validation dataset reaffirmed key biological themes observed in the primary analysis, reinforcing the robustness and biological relevance of the identified CD subtypes. Cluster 1 in both tissue datasets consistently reflected a phenotype driven by adaptive immune activation, marked by enhanced lymphocyte differentiation and B cell signalling, accompanied by suppressed oxidative phosphorylation, suggesting a metabolic trade-off to support heightened immune activity. Such immune-metabolic interplay has been previously noted in active inflammatory states of IBD, where mitochondrial dysfunction parallels immune escalation.^73,74^ Meanwhile, Cluster 2 in the validation data revealed a distinct metabolic axis centred on lipid processing, peroxisomal function and oxidative balance, which, while partially overlapping with the metabolic stress pathways identified in the primary cohort, diverged by showing marked suppression of immune-related pathways. This supports emerging evidence that lipid metabolism and peroxisome biology play critical roles in shaping intestinal immune tolerance and may define less inflammatory but metabolically perturbed CD phenotypes.^73^ Cluster 3 in both datasets demonstrated a convergence around immune cell proliferation and cytokine activity, aligned with rapid immune turnover and heightened cell cycle signalling, further corroborating the inflammatory nature of this subtype.^74^

Notably, the recurrence of immune activation, metabolic reprogramming and mitochondrial involvement across datasets underscores immune-metabolic coupling as a fundamental axis of CD heterogeneity. This is consistent with prior multi-omics IBD studies that have highlighted energy metabolism, cytokine signalling and adaptive immune responses as key contributors to disease stratification.^75,76^ These genes are frequently implicated in intestinal fibrosis, epithelial-to-mesenchymal transition, chromatin remodelling and metabolic reprogramming, which are hallmark features in IBD progression and treatment resistance.^77^

Our clustering analysis of UC transcriptomic subtypes on GSE235236 revealed limited reproducibility. The presence of a single-sample *k*-means cluster indicated likely outlier effects, reflecting limitations in sample size and heterogeneity. Additionally, the overlapping consensus clusters lacked distinct separation, suggesting weak subgroup stratification and low robustness of the clustering solution. These findings highlight the challenges posed by current public datasets, including insufficient power and variability in data quality, which hinder robust validation of UC molecular subtypes. A major limiting factor for the cohort was the small number of treatment-naïve adult UC samples available for analysis.

Although the primary dataset allowed direct molecular profiling at the site of inflammation, its clinical utility is limited by the invasiveness of endoscopic tissue sampling. The use of blood transcriptome data for validation was guided by the framework established by Argmann et al.^37^ who first developed a biopsy-derived molecular inflammation score and subsequently introduced a circulating molecular inflammation score using paired blood samples. Their work demonstrated that peripheral blood gene expression signatures could accurately reflect mucosal inflammation, offering a less invasive and more practical tool for routine monitoring and stratification of IBD patients. Following a similar rationale, our validation strategy leveraged the biological complementarity of these two sample types: tissue for site-specific molecular signals, and blood for systemic, accessible biomarkers. This enabled us to assess the cross-tissue reproducibility of transcriptomic clusters, strengthen the biological relevance of our findings and explore their translational potential in non-invasive diagnostics. Importantly, the blood-based validation supports the feasibility of applying our subtyping framework in real-world clinical settings, where repeated tissue sampling is often not viable.

## Limitations of the study

The analysis relied on unsupervised ML, which, while effective for subtype identification, is highly sensitive to parameter selection. For instance, in *k*-means clustering, parameters such as the number of clusters (*k*) must be predefined. Selecting the optimal *k* is critical, as an inappropriate value can lead to overfitting or underfitting of the data, resulting in clusters that do not accurately represent the underlying biological subtypes. Unsupervised algorithms require careful tuning of parameters, and different choices can yield varying results, especially in high-dimensional data where multiple solutions may appear valid. While the study utilised a substantial tissue biopsy dataset and was verified with a blood biopsy dataset for both UC and CD, and a tissue biopsy dataset for CD, future validation using additional larger transcriptome datasets and independent intestinal tissue cohorts for UC would be advantageous to confirm the robustness of the subtype classification. Furthermore, the inclusion of SHAP (SHapley Additive exPlanations) feature selection could enhance the analysis by identifying the most important genes driving the clustering process.^78^ Microbiome, metabolome-based analyses in IBD, combined with AI-driven studies, could further improve disease prediction and aid in developing personalised diets and treatments for patients with IBD.^20,79–81^ Although this study was focused on classifying UC and CD, inflammatory bowel disease unclassified (IBDU) represents a clinically relevant group that remains challenging to diagnose and manage. As IBDU cases often share overlapping features with both UC and CD, future work applying this transcriptomic subtyping framework may help clarify disease classification in these patients and support more personalised treatment strategies.

## Future work

To address the limitation of stringent gene cut-offs and large transcriptomic datasets in this study, future analyses could involve replicating the approach with additional clustering algorithms such as density-based clustering methods^67^ and spectral clustering,^80^ as well as predictive modelling applied to multi-omics datasets. Incorporating single-cell RNA (scRNA)-seq from adult IBD patients would allow for a more granular exploration of gene expression at the individual cell level, compared to bulk RNA sequencing.^82^ This approach could provide deeper insights into cellular heterogeneity and rare cell types, addressing the drawbacks of bulk RNA-seq.

Additionally, integrating autoencoders, a class of artificial neural networks used for unsupervised learning, into the analysis pipeline could enhance feature extraction and dimensionality reduction in high-dimensional gene expression data. Autoencoders have been effectively applied in clustering scRNA-seq data, improving the identification of cell types and states. For instance, the scMAE (single-cell Masked Autoencoder) model has demonstrated success in clustering scRNA-seq data by learning robust representations of gene expression profiles.^83^ Incorporating autoencoders could enhance the analysis by capturing complex, non-linear relationships within the data, leading to more accurate patient stratification and a deeper understanding of the molecular mechanisms underlying IBD.

### Targeting drugs to clusters

The identified clusters offer a foundation for developing cluster-specific therapeutic strategies. By characterising the unique molecular and clinical features of each cluster, drugs can be targeted to address the predominant pathways and mechanisms at play. Clusters enriched with pathways related to autophagy regulation, RNA modification, immune dysregulation or cytoskeletal organisation could guide the repurposing of existing therapies (e.g. autophagy modulators, JAK inhibitors) or the development of new therapeutics based on novel markers (e.g. *IL17RA, RXRA, ErbB2*).

By aligning therapeutic strategies with the molecular and clinical characteristics of each cluster, this approach could significantly enhance the personalisation of IBD treatment, leading to better outcomes for patients.

## Conclusion

This study categorises UC and CD into three transcriptomic subtypes, each defined by unique transcriptomic profiles and associated molecular pathways. For UC, the subtypes are characterised by: (1) high RNA processing and protein synthesis activity, (2) upregulation of autophagy and cellular signalling pathways and (3) emphasis on cytoskeletal organisation and intracellular trafficking. Similarly, for CD, the subtypes include (1) a focus on cytoskeletal dynamics and viral response pathways, (2) heightened protein synthesis and stress responses and (3) strong emphasis on epigenetic regulation and cellular stability. These subtypes not only reflect underlying biological heterogeneity but also demonstrate significant correlations with clinical indicators of disease activity. In both UC and CD, Cluster 3 was significantly enriched for patients exhibiting moderate to severe endoscopic inflammation, while Cluster 1 showed a predominance of inactive or mild disease, as assessed by validated endoscopic scoring systems. Moreover, subtype distribution was strongly associated with anatomical disease location, with distinct transcriptomic signatures observed in colonic versus ileal involvement. Validation across tissue (intestinal biopsies) and blood-derived transcriptomic datasets reinforces the robustness and translational value of these subtypes. Through detailed analysis, meaningful patterns associated with IBD were identified, along with key genes contributing to the subtyping, thereby enhancing the understanding of the disease’s complex pathogenesis. These findings have the potential to guide the development of novel therapeutic strategies and personalised medicine tailored to patients with specific IBD subtypes, moving beyond the conventional treatments for UC and CD. Such advancements could significantly improve patient outcomes by enabling precise and targeted therapeutic approaches.

## Supplemental Material

sj-docx-1-tag-10.1177_17562848251362391 – Supplemental material for Identifying inflammatory bowel disease subtypes: a comprehensive exploration of transcriptomic data and machine learning-based approaches Supplemental material, sj-docx-1-tag-10.1177_17562848251362391 for Identifying inflammatory bowel disease subtypes: a comprehensive exploration of transcriptomic data and machine learning-based approaches by Niyati Saini and Animesh Acharjee in Therapeutic Advances in Gastroenterology

## Declarations

**Ethics approval and consent to participate:** Ethics approval and consent to participate is not required.

**Consent for publication:** Not applicable.

**Availability of data and materials:** All scripts supporting the findings of this study are publicly available on Figshare at https://doi.org/10.6084/m9.figshare.28200200.v5. Additional Supplemental Materials, including detailed methodologies and extended data tables, have been included as Supplemental Files and can be accessed alongside the main manuscript.
