# Supplementary material for: Identifying inflammatory bowel disease subtypes: a comprehensive exploration of transcriptomic data and machine learning-based approaches
Source: Ther Adv Gastroenterol. 2025 Aug 12;18:17562848251362391. doi: 10.1177/17562848251362391 (PMC12344326; doi:10.1177/17562848251362391)
Supplement: sj-docx-1-tag-10.1177_17562848251362391 – Supplemental material for Identifying inflammatory bowel disease subtypes: a comprehensive exploration of transcriptomic data and machine learning-based approaches [file sj-docx-1-tag-10.1177_17562848251362391.docx]

**Supplementary Files**

**Identifying Inflammatory Bowel Disease Subtypes: A Comprehensive Exploration of Transcriptomic Data and Machine Learning-Based Approaches**

Niyati Saini ^1^, Animesh Acharjee ^1,2,3*^

1. Cancer and Genomic Sciences, School of Medical Sciences, College of Medicine and Health, University of Birmingham, B15 2TT, UK
2. Institute of Translational Medicine, University Hospitals Birmingham NHS Foundation Trust, B15 2TT, Birmingham, UK
3. Centre for Health Data Research, University of Birmingham, B15 2TT, UK

***Correspondence**

Dr. Animesh Acharjee

Cancer and Genomic Sciences

University of Birmingham, B15 2TT, Birmingham, UK

**E-mail:** [a.acharjee@bham.ac.uk](mailto:a.acharjee@bham.ac.uk)

Table S1. Tabulated the STROBE Checklist for Reporting of Cohort Studies. Each item is mapped to the relevant section and page number of the manuscript.

|  | Item No | Recommendation | Page No |
| --- | --- | --- | --- |
| **Title and abstract** | 1 | (*a*) Indicate the study’s design with a commonly used term in the title or the abstract | 1 |
|  |  | (*b*) Provide in the abstract an informative and balanced summary of what was done and what was found | 2-3 |
| Introduction | | | |
| Background/rationale | 2 | Explain the scientific background and rationale for the investigation being reported | 4-5 |
| Objectives | 3 | State specific objectives, including any prespecified hypotheses | 5-6 |
| Methods | | | |
| Study design | 4 | Present key elements of study design early in the paper | 6-7 |
| Setting | 5 | Describe the setting, locations, and relevant dates, including periods of recruitment, exposure, follow-up, and data collection | 7 |
| Participants | 6 | (*a*) Give the eligibility criteria, and the sources and methods of selection of participants. Describe methods of follow-up | 7-8 |
|  |  | (*b*) For matched studies, give matching criteria and number of exposed and unexposed | NA |
| Variables | 7 | Clearly define all outcomes, exposures, predictors, potential confounders, and effect modifiers. Give diagnostic criteria, if applicable | 7-9 |
| Data sources/ measurement | 8* | For each variable of interest, give sources of data and details of methods of assessment (measurement). Describe comparability of assessment methods if there is more than one group | 7-9 |
| Bias | 9 | Describe any efforts to address potential sources of bias | 6,22 |
| Study size | 10 | Explain how the study size was arrived at | 6-7, Figure S1 |
| Quantitative variables | 11 | Explain how quantitative variables were handled in the analyses. If applicable, describe which groupings were chosen and why | 8-9 |
| Statistical methods | 12 | (*a*) Describe all statistical methods, including those used to control for confounding | 8-10 |
|  |  | (*b*) Describe any methods used to examine subgroups and interactions | 10-11 |
|  |  | (*c*) Explain how missing data were addressed | 7-8 |
|  |  | (*d*) If applicable, explain how loss to follow-up was addressed | NA |
|  |  | (*e*) Describe any sensitivity analyses | NA |
| Results | | |  |
| Participants | 13* | (a) Report numbers of individuals at each stage of study—eg numbers potentially eligible, examined for eligibility, confirmed eligible, included in the study, completing follow-up, and analysed | 7, Figure S1 |
|  |  | (b) Give reasons for non-participation at each stage | Figure S1 |
|  |  | (c) Consider use of a flow diagram | Figure S1 |
| Descriptive data | 14* | (a) Give characteristics of study participants (eg demographic, clinical, social) and information on exposures and potential confounders | 7, Table 1 |
|  |  | (b) Indicate number of participants with missing data for each variable of interest | NA |
|  |  | (c) Summarise follow-up time (eg, average and total amount) | NA |
| Outcome data | 15* | Report numbers of outcome events or summary measures over time | 12-14 |
| Main results | 16 | (*a*) Give unadjusted estimates and, if applicable, confounder-adjusted estimates and their precision (eg, 95% confidence interval). Make clear which confounders were adjusted for and why they were included | NA |
|  |  | (*b*) Report category boundaries when continuous variables were categorized | NA |
|  |  | (*c*) If relevant, consider translating estimates of relative risk into absolute risk for a meaningful time period | NA |
| Other analyses | 17 | Report other analyses done—eg analyses of subgroups and interactions, and sensitivity analyses | NA |
| Discussion |  |  |  |
| Key results | 18 | Summarise key results with reference to study objectives | 12-21 |
| Limitations | 19 | Discuss limitations of the study, taking into account sources of potential bias or imprecision. Discuss both direction and magnitude of any potential bias | 29 |
| Interpretation | 20 | Give a cautious overall interpretation of results considering objectives, limitations, multiplicity of analyses, results from similar studies, and other relevant evidence | 21-28, 30-32 |
| Generalisability | 21 | Discuss the generalisability (external validity) of the study results | 21-23 |
| Other information |  |  |  |
| Funding | 22 | Give the source of funding and the role of the funders for the present study and, if applicable, for the original study on which the present article is based | 32 |

Table S2: Tabulated the list of tools and packages for project documentation and ensuring reproducibility. This detailed table shows the summary of all the necessary tools and packages used in this analysis.

| Tools and Software | Task | Version |
| --- | --- | --- |
| R | Statistical computing | 4.4.1 |
| Biocmanager | To install and update Bioconductor packages | 1.30.23 |
| Limma | Differential Gene Expression (DGE) Analysis | 3.60.4 |
| Limma-voom | Data transformation | 3.60.4 |
| data.table | Data manipulation | 1.15.4 |
| GEO query | Data retrieval | 2.72.0 |
| edgeR | Data normalization | 4.2.1 |
| dplyr | Data manipulation | 1.1.4 |
| Enhanced Volcano | Data visualization | 1.22.0 |
| pheatmap | Data visualization | 1.0.12 |
| Complex heatmap | Advanced data visualization | 2.20.0 |
| Org.Hs.eg.db | Mapping | 3.19.1 |
| Annotation Dbi | Annotation Database Interface | 1.66.0 |
| ggplot2 | Creating detailed and customizable plots | 3.5.1 |
| ConsensusClusterPlus | Consensus clustering analysis. | 1.68.0 |
| factoextra | Visualizing results from multi-variate (clustering) analysis | 1.0,7 |
| dendextend | Enhanced dendrograms | 1.17.1 |
| ggrepel | To add non-overlapping text labels and generate clear plots | 0.9.5 |
| ggraph | Generating graphs and networks | 2.2.1 |
| igraph | For network analysis and visualization | 2.0.3 |
| ggpubr | Enhancing ggplot2 visualizations | 0.6.0 |
| tidyverse | Data manipulation and cleaning | 2.2.0 |
| ReactomePA | For pathway enrichment analysis (Reactome pathways) | 1.48.0 |
| ClusterProfiler | Functional Enrichment analysis and visualization. | 4.12.2 |
| WGCNA | Constructing and analyzing gene co-expression networks | 1.72-5 |
| enrichplot | GSEA visualization | 1.24.2 |
| DOSE | Enrichment analysis | 3.30.2 |
| GOSemSim | Enrichment analysis | 2.30.0 |

Table S3: Tabulated the results from statistical data analysis on primary dataset for linking clinical data attributes with subtypes identified.

| **Attribute** | **Test type** | **Dataset** | **X squared F value** | **Degree of freedom** | **P value** | **Significant** |
| --- | --- | --- | --- | --- | --- | --- |
| **IBD clinician’s measure** | Chi- squared test | UC | 5.14 | 2 | 7.642e-02 | No |
| **IBD clinician’s measure** | Chi- squared test | CD | 5.20 | 2 | 7.404e-02 | No |
| **IBD endo severity** | Chi- squared test | UC | 25.61 | 6 | 2.630e-04 | Yes |
| **IBD endo severity** | Chi- squared test | CD | 17.70 | 6 | 7.006e-03 | Yes |
| **Gender** | Chi- squared test | UC | 2.69 | 2 | 2.593e-01 | No |
| **Gender** | Chi- squared test | CD | 2.46 | 2 | 2.919e-01 | No |
| **Region** | Chi- squared test | UC | 84.24 | 12 | 6.363e-13 | Yes |
| **Region** | Chi- squared test | CD | 57.43 | 12 | 6.595e-08 | Yes |
| **Disease type (inflamed or non-inflamed)** | Chi- squared test | UC | 20.44 | 2 | 3.642e-05 | Yes |
| **Disease type**  **(inflamed or non-inflamed)** | Chi- squared test | CD | 5.20 | 2 | 7.411e-02 | No |
| **Age** | ANOVA | UC | 10.38 | 2 | 3.540e-05 | Yes |
| **Age** | ANOVA | CD | 1.25 | 2 | 2.850e-01 | No |

Table S4: Number of samples and significant genes (FDR< 0.001) in each CD cluster of GSE137344 upon DGE analysis.

| **Cluster Comparison** | **Number of Samples**  **(CD Cluster)** | **Significant genes**  **(CD)** |
| --- | --- | --- |
| Cluster 1 vs others | 43 | 958 |
| Cluster 2 vs others | 40 | 2044 |
| Cluster 3 vs others | 29 | 1147 |


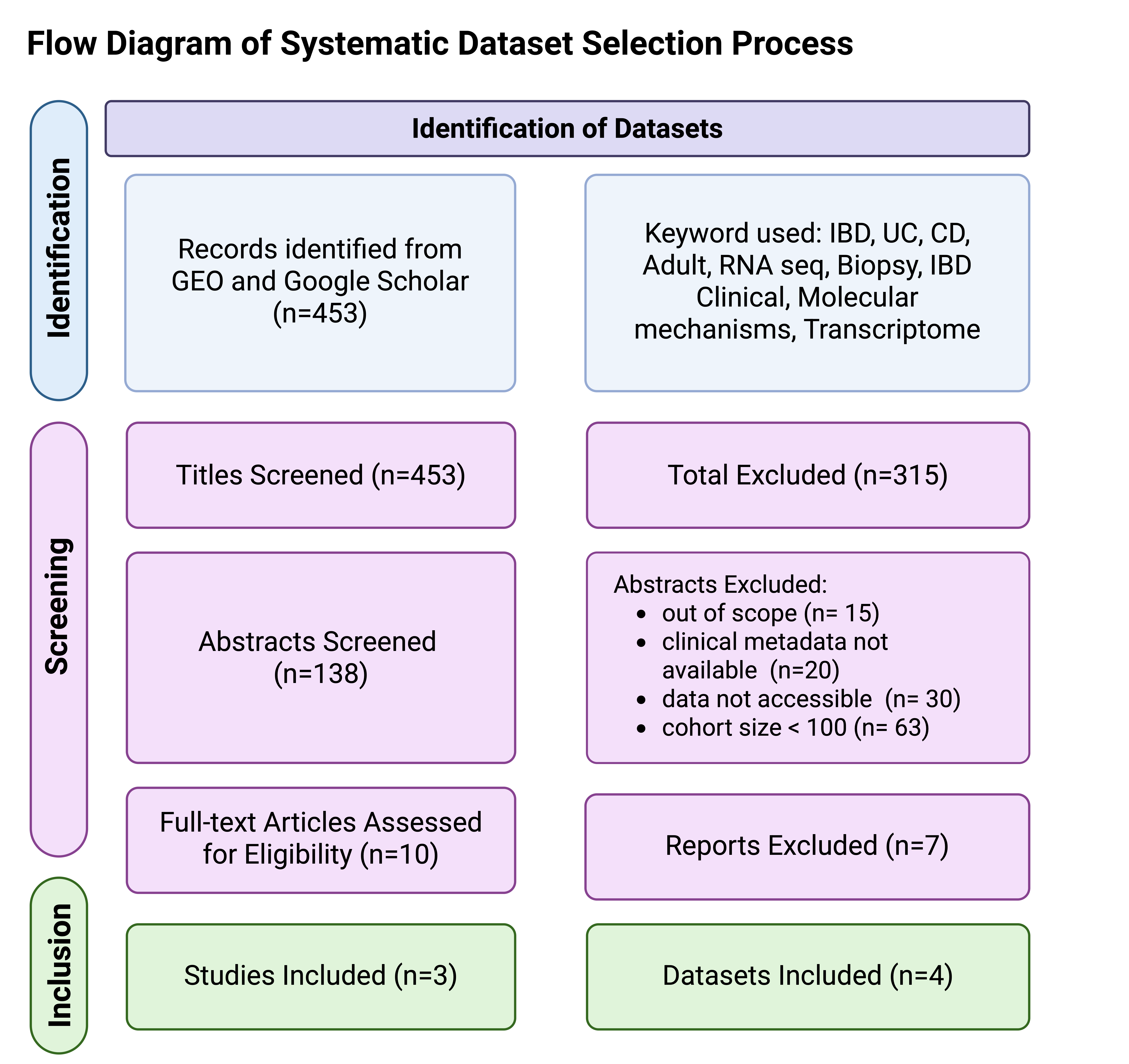


Figure S1: Flow diagram of systematic dataset selection process. This figure outlines the systematic search and selection of transcriptomic studies and datasets from GEO, SRA and Google Scholar. Based on predefined criteria, including cohort type, sample size, metadata availability, and data format, four datasets were selected for analysis. Out of which, two datasets were selected as primary datasets from a single eligible study: GSE193677 (tissue) and GSE186507 (blood).


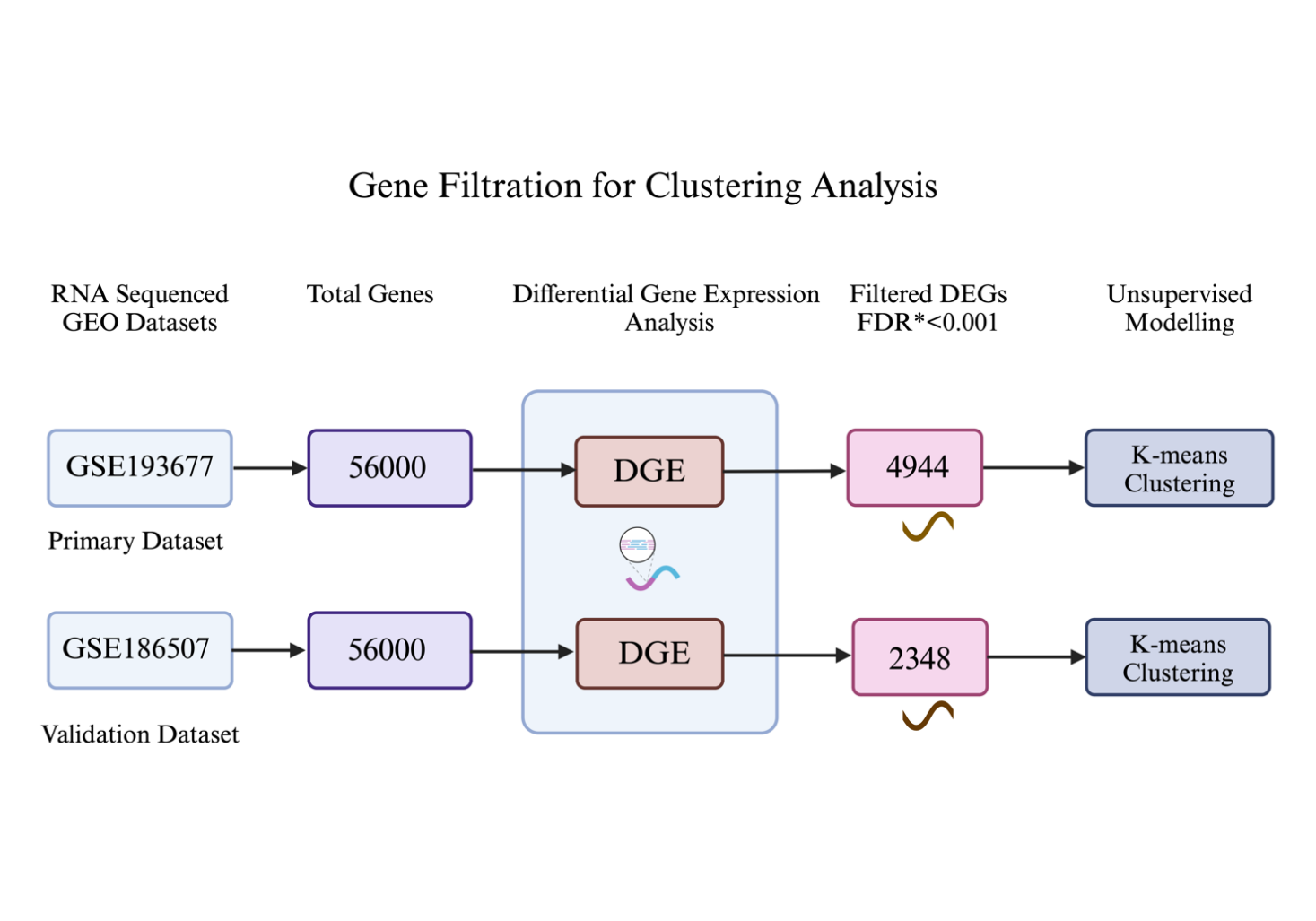


Figure S2: Differential gene expression (DGE*) analysis for gene filtration. Upon DGE analysis using limma, significant genes with false discovery rate (FDR*) < 0.001 selected for clustering analysis.


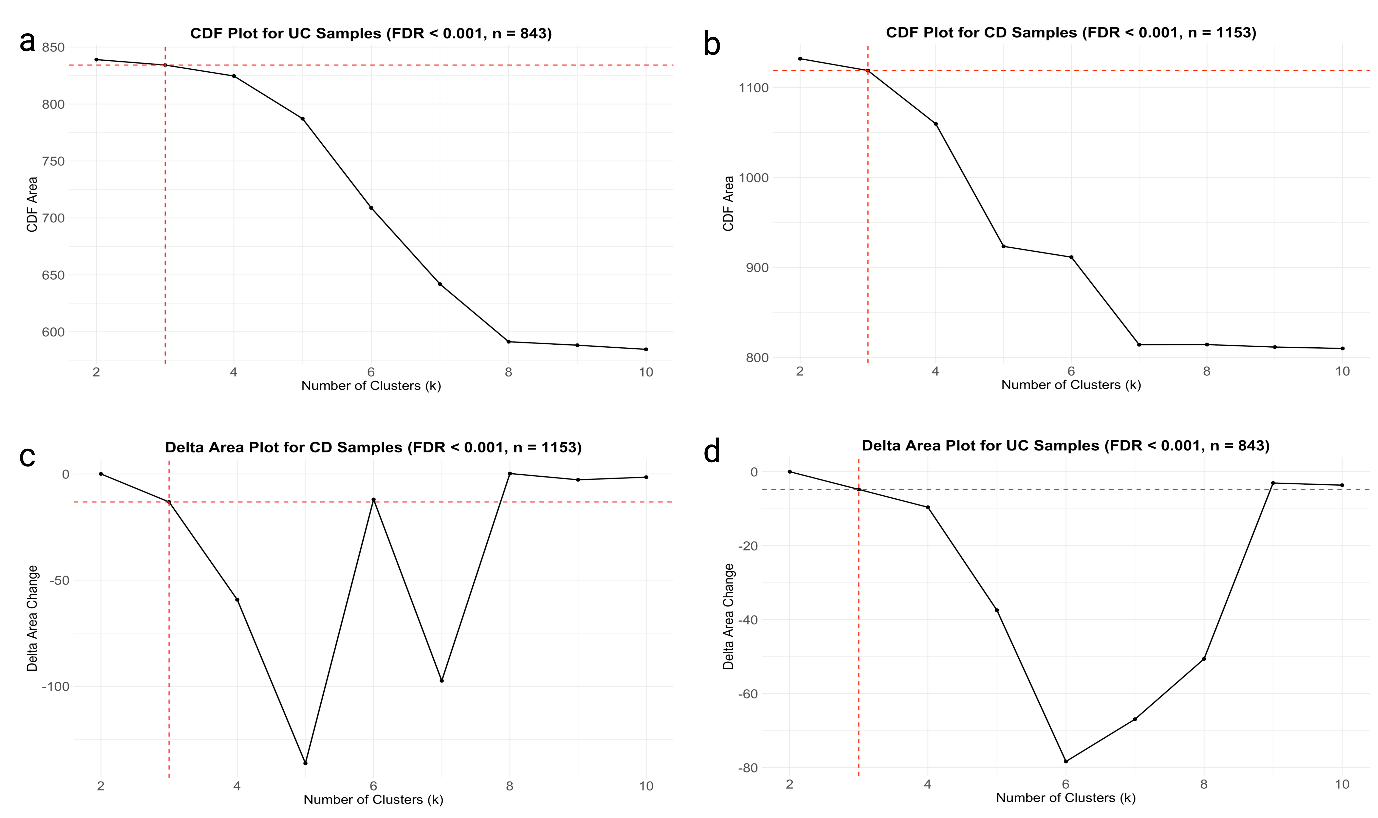


Figure S3: Consensus clustering evaluation for primary intestinal biopsy dataset. a,b. Empirical cumulative distribution function (CDF) plots for Ulcerative Colitis (UC, n = 843) and Crohn’s Disease (CD, n = 1153) samples across k = 2 to 10 clusters. A plateauing of the CDF curve after k = 3 indicates relative cluster stability. c,d. Corresponding delta area plots show the change in the area under the CDF curve between successive k-values. The largest drop occurs after k = 3, further supporting k = 3 as the optimal cluster solution for both UC and CD subgroups.


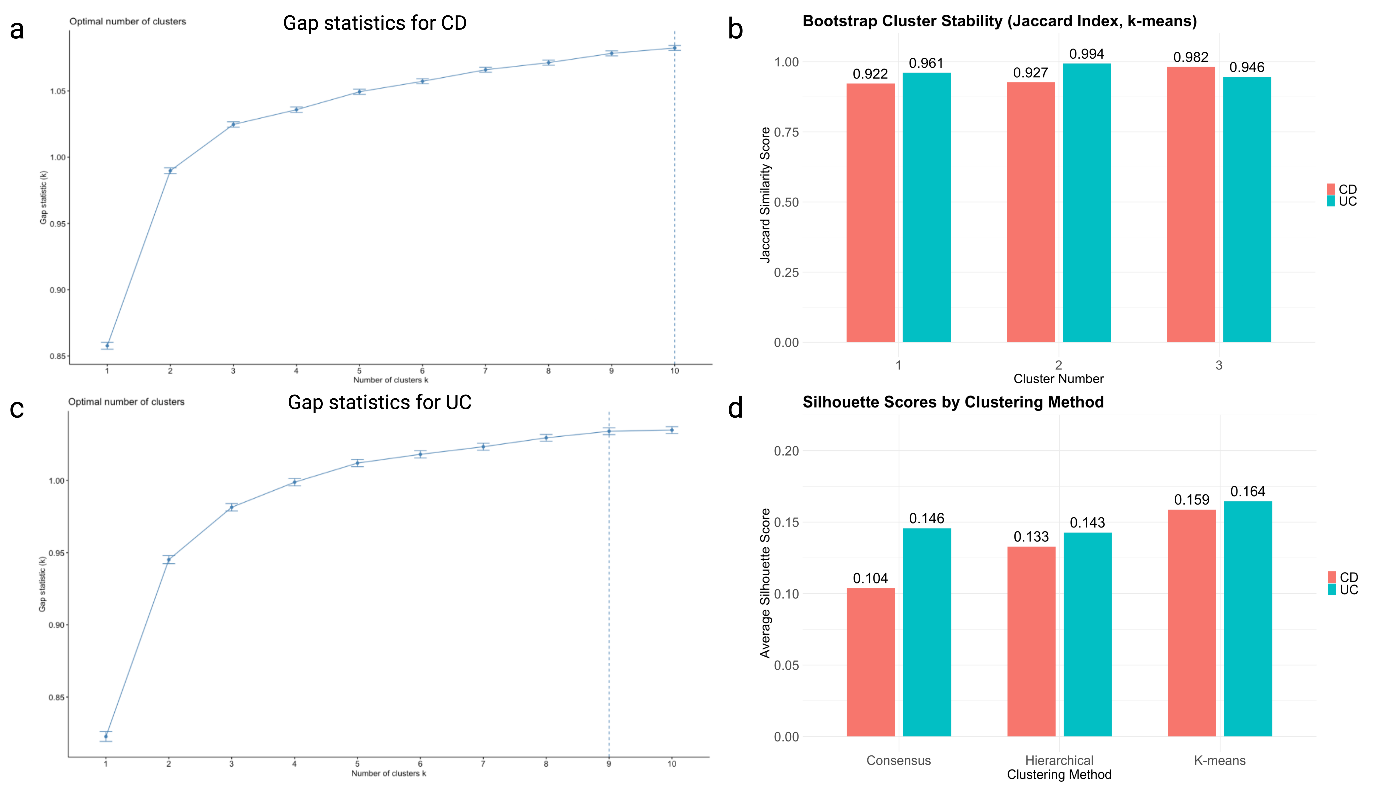


Figure S4: Internal validation metrics for primary intestinal biopsy dataset. a, c. Gap statistic plots for CD and UC samples showing increasing separation up to k = 9, but optimal clustering stability observed near k = 3 as corroborated by other methods. b. Jaccard index plots from bootstrap resampling confirm very stable clustering for K-means clusters (Cluster 1: CD = 0.922, UC = 0.961). d. Silhouette scores show slightly better inter-cluster separation in k-means (UC: 0.164; CD: 0.159), suggesting this as the most appropriate clustering method in this dataset.


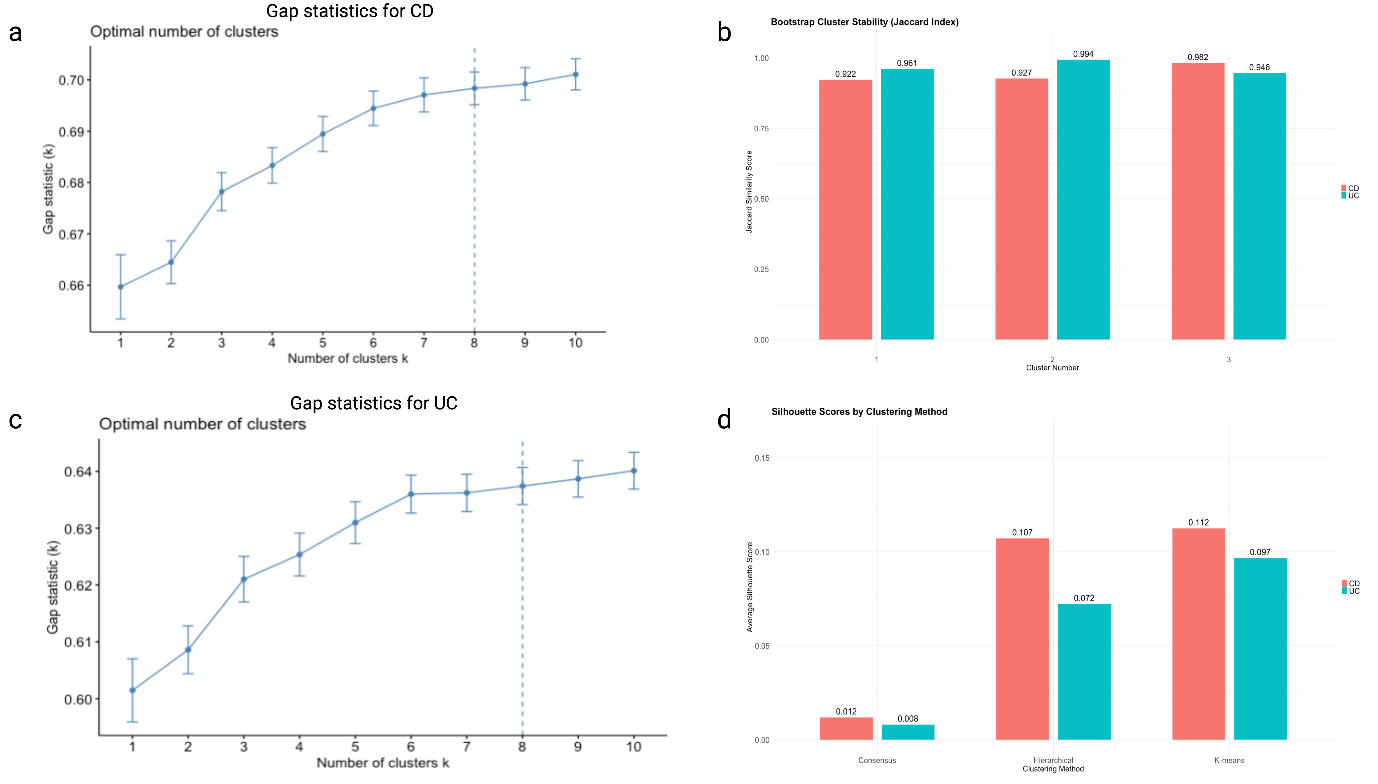


Figure S5: Internal validation metrics for the blood dataset. a, c. Gap statistic plots for CD and UC blood samples showing increasing separation up to k = 8, but optimal clustering stability observed near k = 3 as corroborated by other methods. b. Bootstrap Jaccard index plots for k-means clustering show moderately stable clusters (0.60–0.75), with Cluster 2 in UC exceeding the 0.85 stability threshold. d. Silhouette score comparison among clustering methods shows highest compactness for k-means (UC: 0.097; CD: 0.112), followed by hierarchical and consensus clustering.


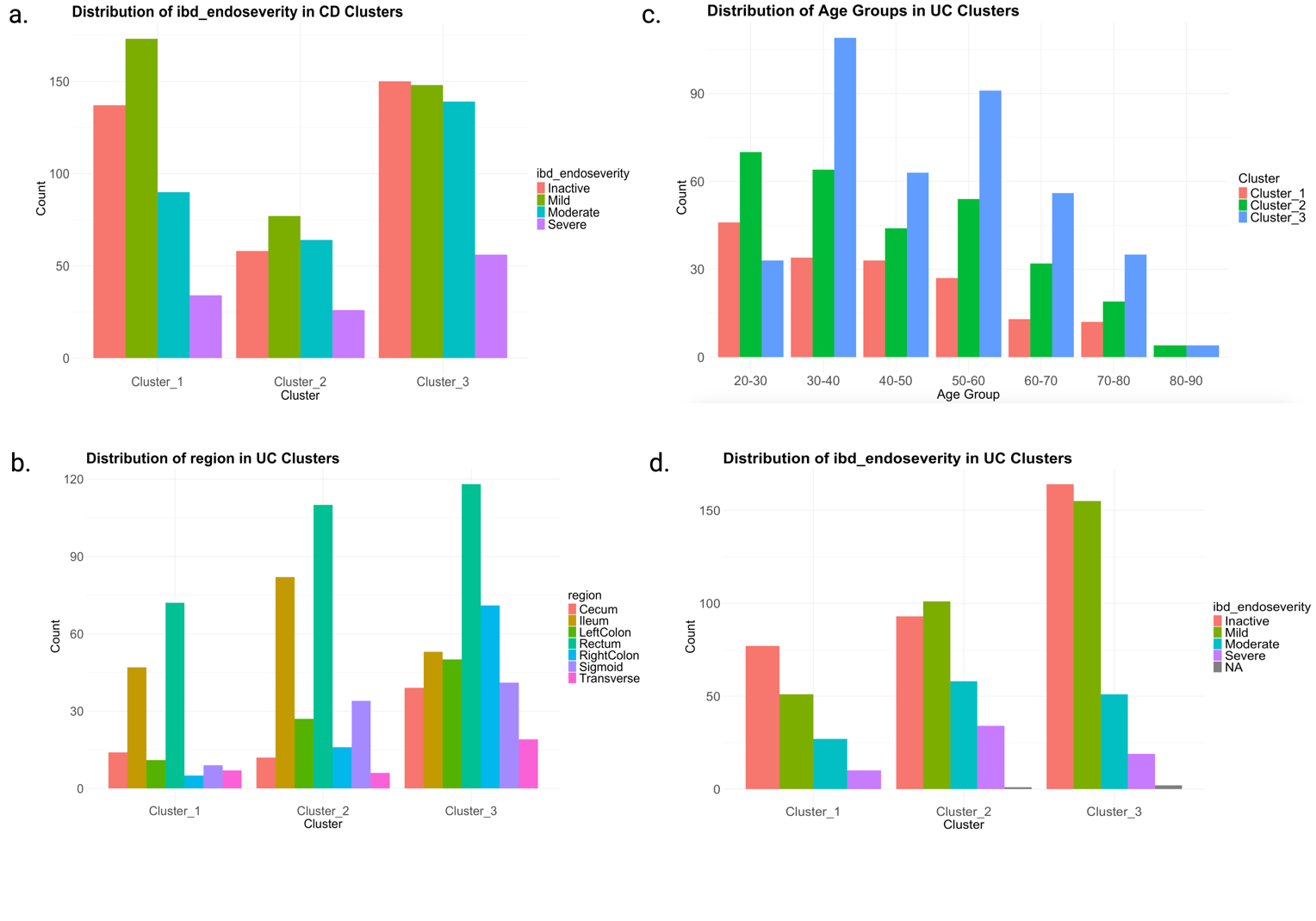


Figure S6: Distribution of Clinical Features Across Clusters in UC and CD in primary dataset. a. Bar plot showing the distribution of IBD endoscopic severity (ibd_endoseverity) across the 3 CD clusters. b. Bar plot illustrating the distribution of anatomical regions affected (region) across the 3 UC clusters. c. Bar plot displaying the distribution of age groups across the UC clusters. d. Bar plot showing the distribution of IBD endoscopic severity (ibd_endoseverity) across UC clusters. Each plot highlights variations in clinical features annotated by clusters, helping to elucidate distinct patterns across UC and CD.


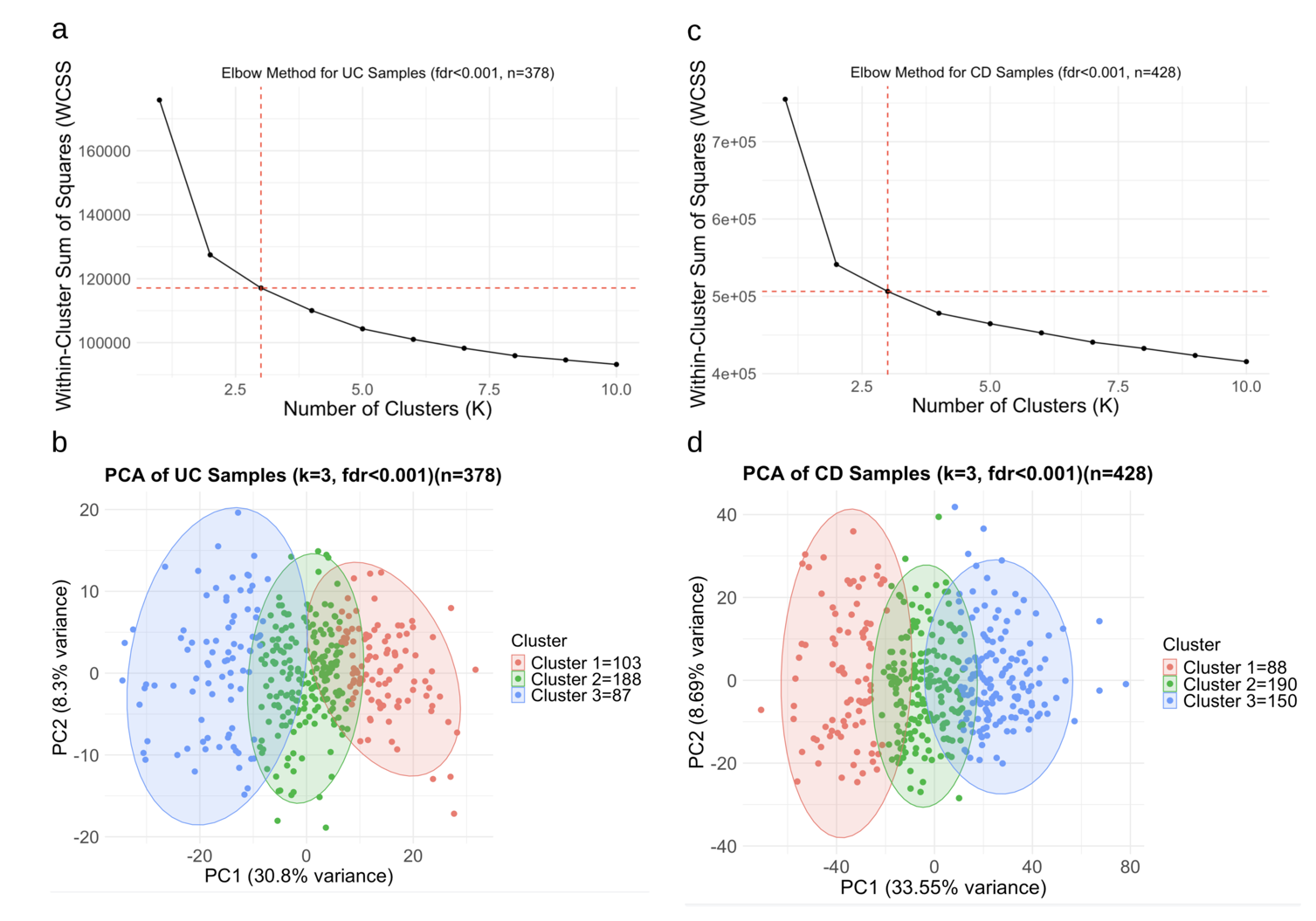


Figure S7: Results from K value selection and clustering analysis on blood validation dataset. a,c. Elbow plot showing k=3 as appropriate K- value for UC and CD datasets respectively. b, d. PCA Plot of K-means Clustering showing 3 clusters within UC and CD samples respectively a, c. X- axis represents the number of clusters (k) and, Y- axis depicts Within-cluster sum of squares (WCSS) and the red dotted line indicates the optimum value of k. b, d. X-axis and Y-axis shows PC1 and PC2 respectively with variance in %.


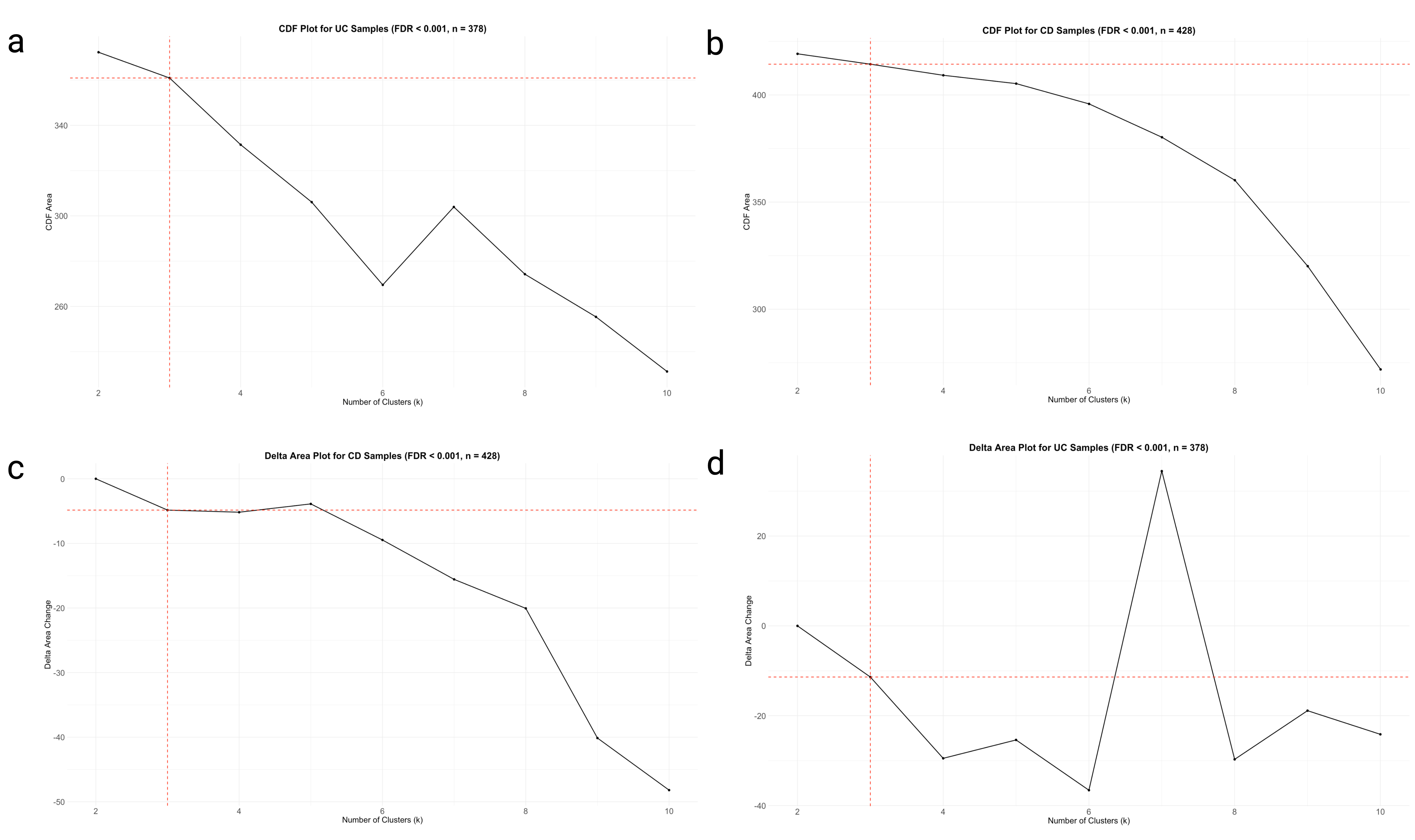


Figure S8: Consensus clustering evaluation for the blood transcriptomic validation dataset. *a*,b. CDF plots of consensus matrices for UC (n = 378) and CD (n = 428) blood samples. c,d. Delta area plots show the relative gain in consensus clustering stability. The k = 3 solution is marked by reduced delta gain beyond this point, indicating optimal clustering. Despite a smaller dataset size than the biopsy data, clustering trends remained consistent.


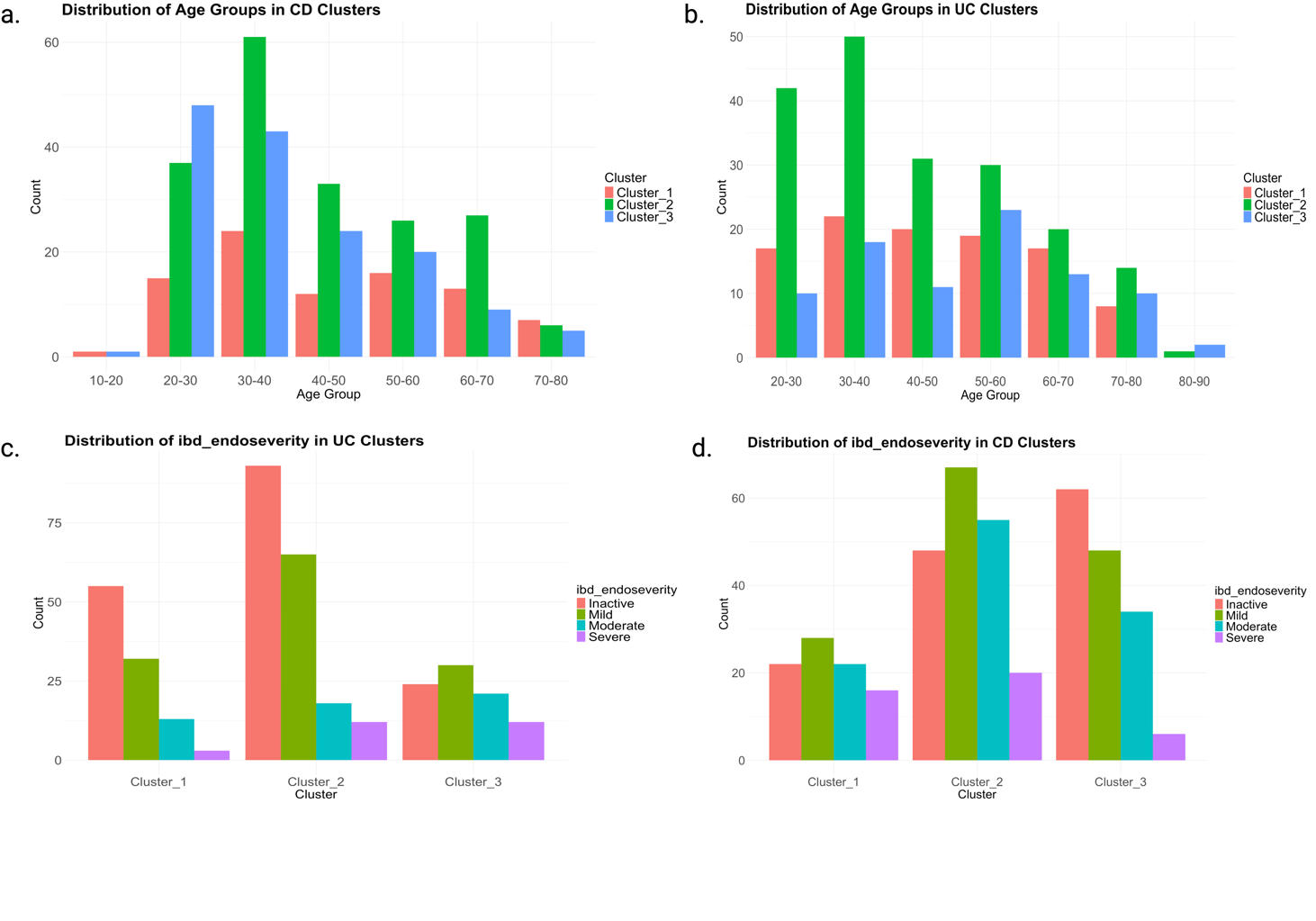


Figure S9: Comparative Distribution of Age Groups and IBD Endoscopic Severity Across UC and CD Clusters in the blood validation dataset. a, b. Bar plot illustrating the distribution of age groups across CD and UC clusters respectively. c, d. Bar plot representing the distribution of IBD endoscopic severity (ibd_endoseverity) across UC and CD clusters respectively.


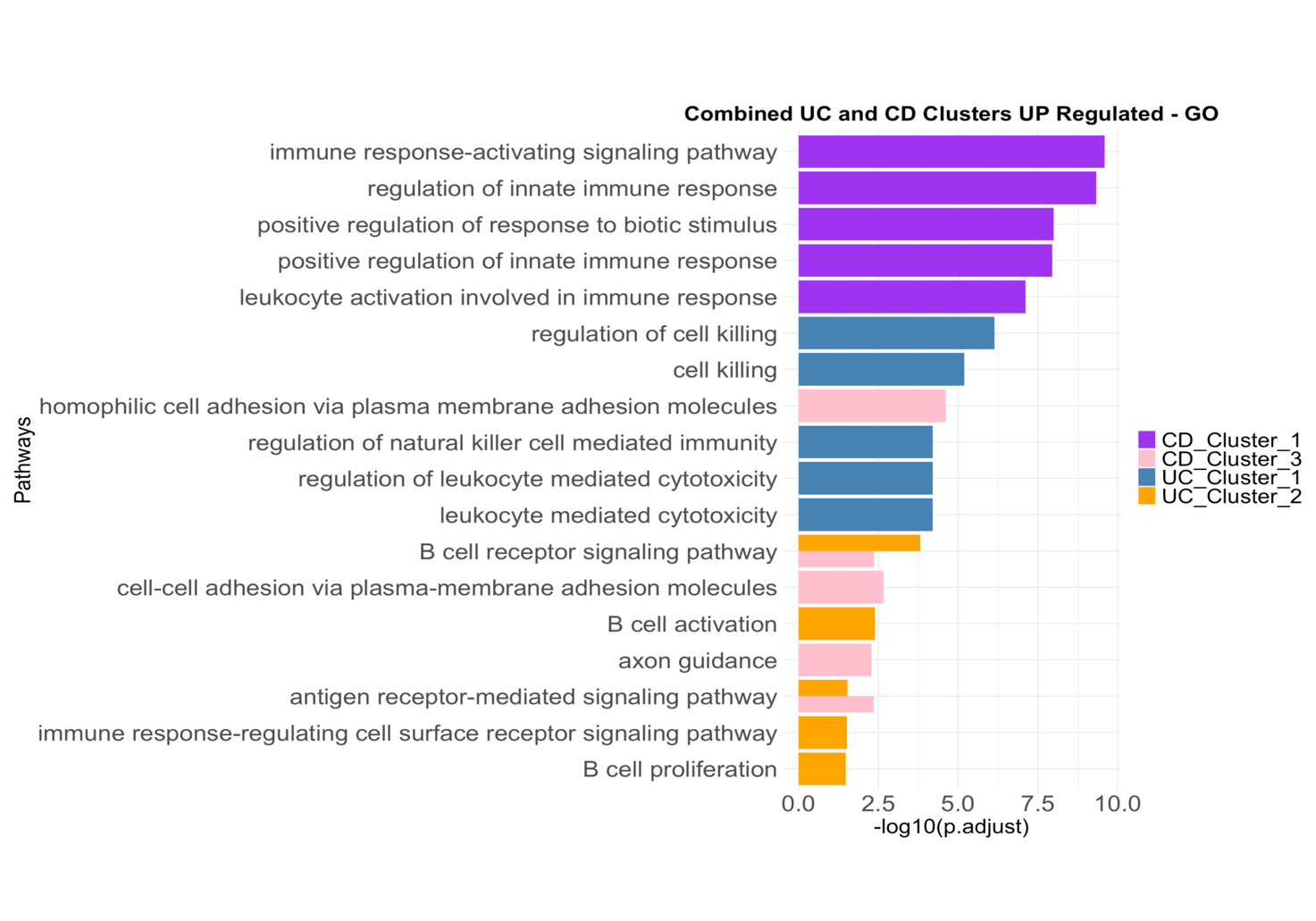


Figure S10: Combined bar plot showing the top enriched upregulated GO pathways across UC and CD clusters in the blood validation dataset.


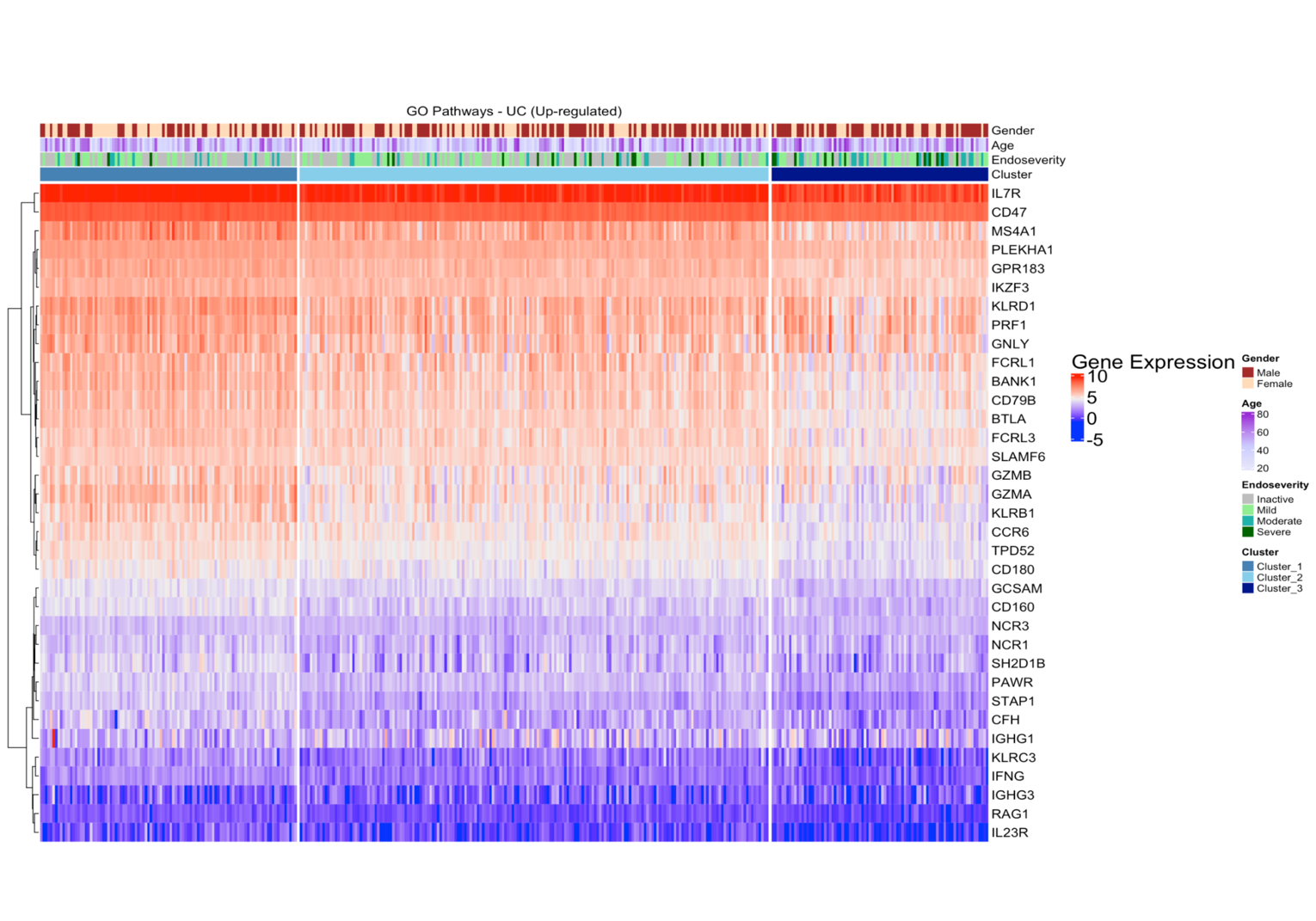


Figure S11: Heatmap of Genes from the Top 5 Upregulated GO Pathways in UC (Blood Validation Dataset). This heatmap visualizes the expression levels of genes from the top 5 upregulated GO pathways in UC clusters within the validation dataset. Samples are annotated by clusters and clinical features, including gender, age, endoscopic severity, and region. The plot highlights the differential expression patterns of genes across clusters, emphasizing their roles in upregulated GO pathways.


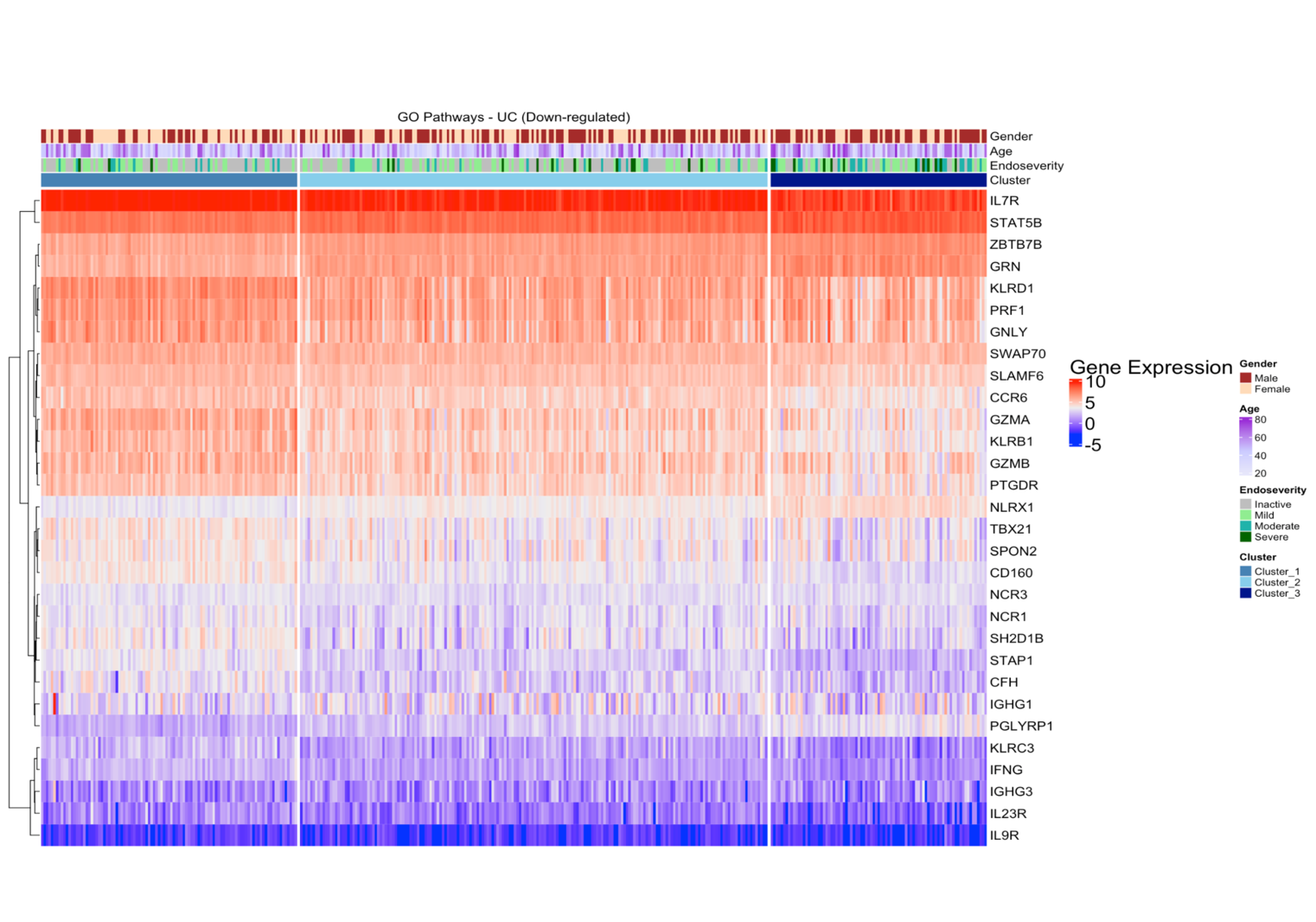

Figure S12: Heatmap of Genes from the Top 5 Downregulated GO Pathways in UC (Blood Validation Dataset). This heatmap visualizes the expression levels of genes from the top 5 downregulated GO pathways in UC clusters within the validation dataset. Samples are annotated by clusters and clinical features, including gender, age, endoscopic severity, and region. The plot highlights the differential expression patterns of genes across clusters, emphasizing their roles in downregulated GO pathways.


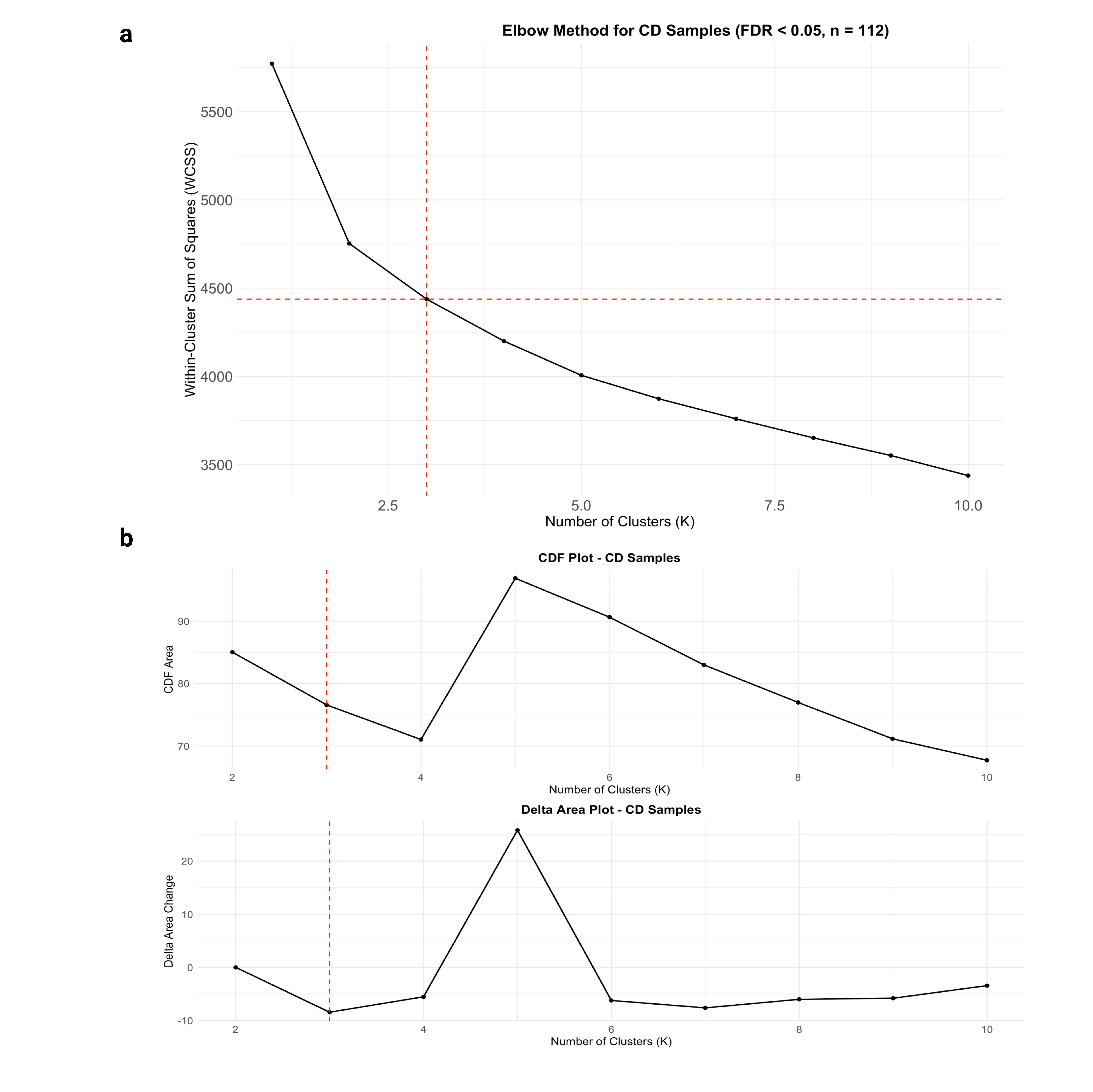


Figure S13. Clustering evaluation of transcriptomic subtypes in the CD validation dataset (GSE137344). a. Elbow plot showing the optimal number of clusters (k = 3), where the x-axis represents the number of clusters and the y-axis indicates the within-cluster sum of squares (WCSS). Red dashed lines mark the chosen k. b. CDF and delta area plots from consensus clustering for CD samples (FDR < 0.05, n = 112), supporting k = 3 as the most stable solution.


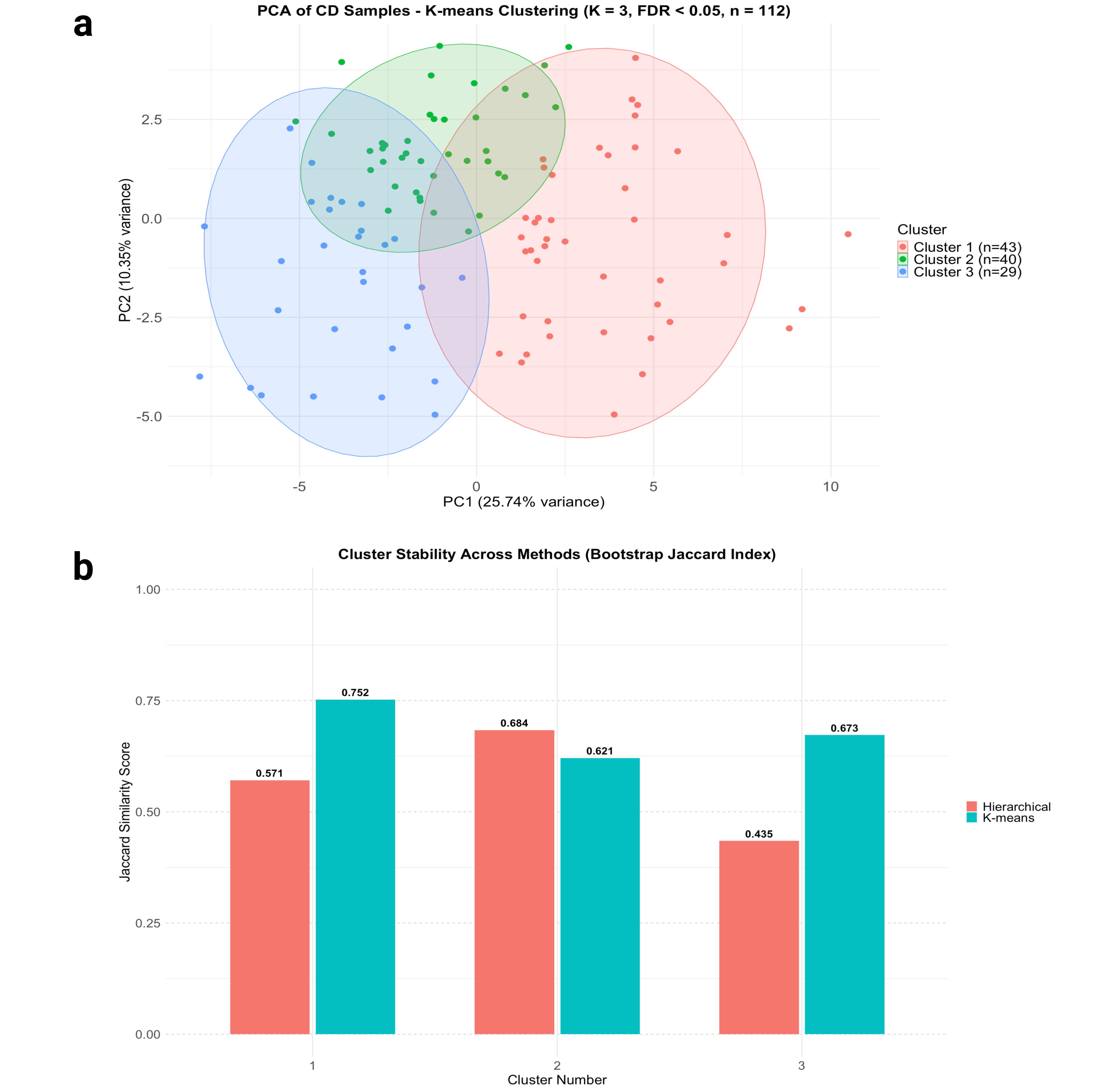


Figure S14. Internal validation of transcriptomic subtypes in the CD validation dataset (GSE137344). a. PCA plot of CD samples clustered by k-means (k = 3), displaying separation across the first two principal components (PC1 and PC2). b. Bar plot showing cluster-wise bootstrap Jaccard similarity scores comparing k-means (blue) and hierarchical clustering (red) methods.


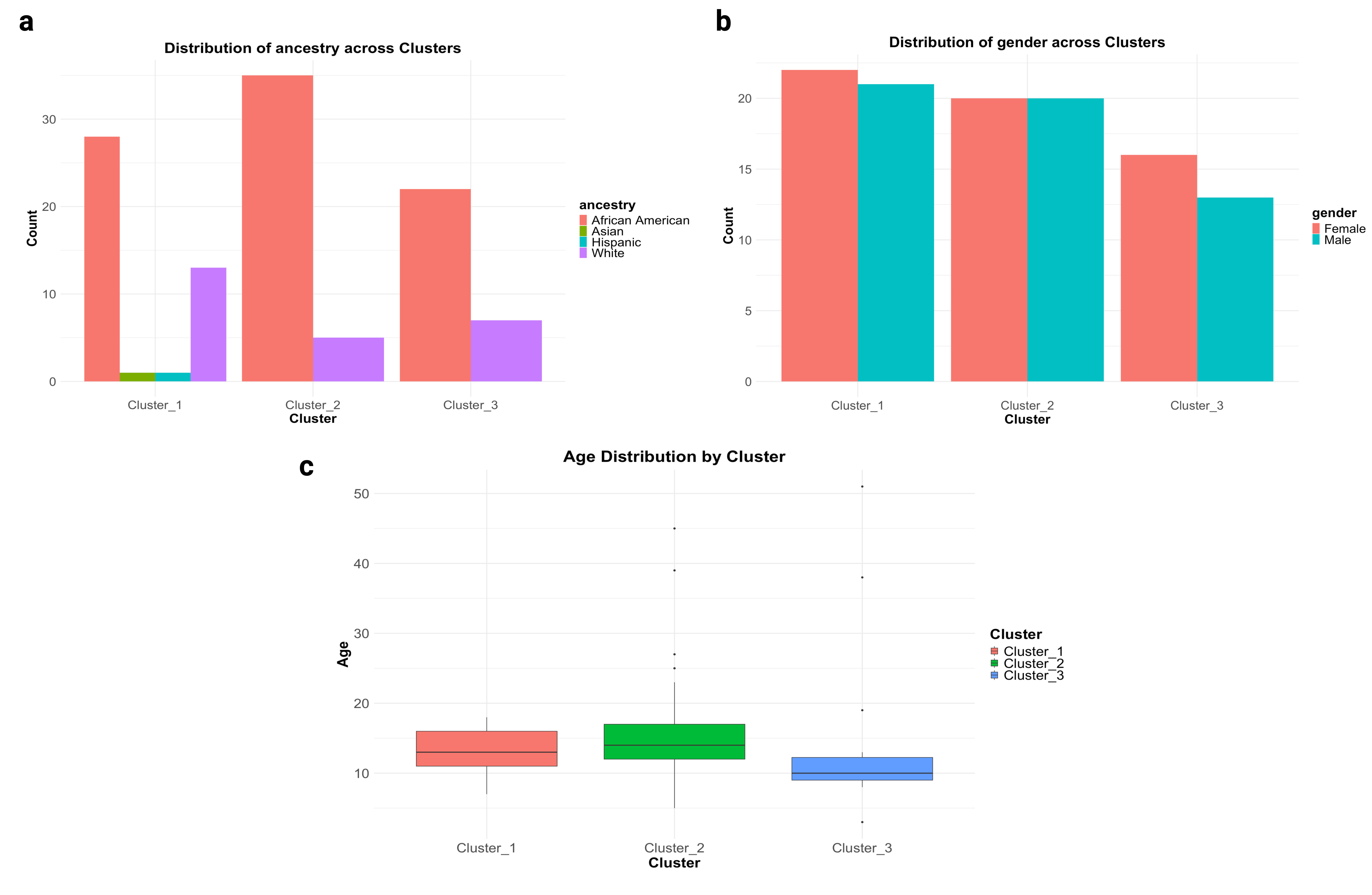


Figure S15: Comparative Distribution of Ancestry, Region and Age groups across CD Clusters in the validation dataset. a. Bar plot illustrating the distribution of ancestry across CD clusters. b. Bar plot representing the distribution of region across CD clusters. c. Bar plot illustrating the distribution of ancestry across CD clusters.


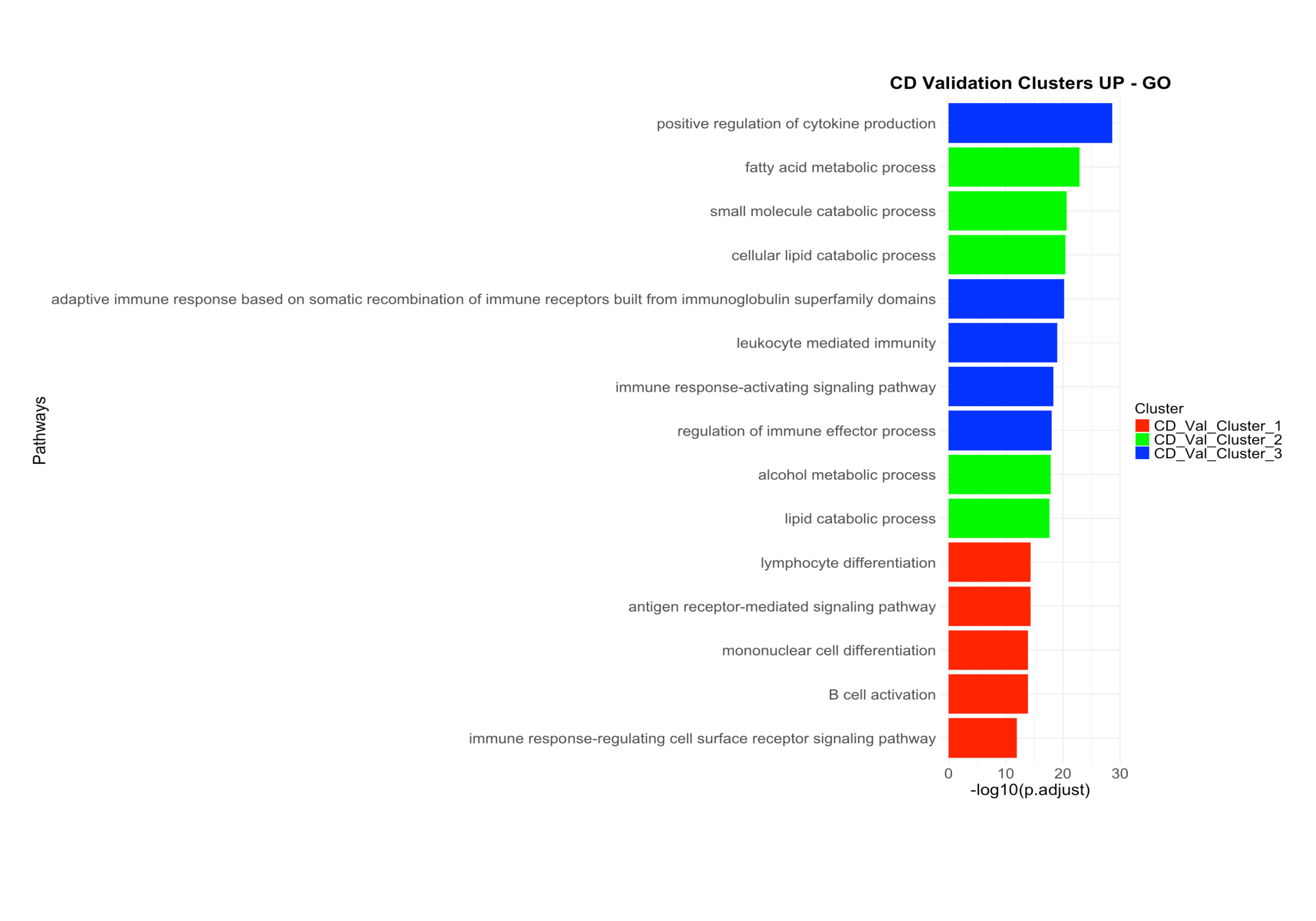


Figure S16: Results from GSEA analysis on GSE137344 CD Validation dataset. Combined bar plot showing the top enriched upregulated GO pathways across CD clusters.


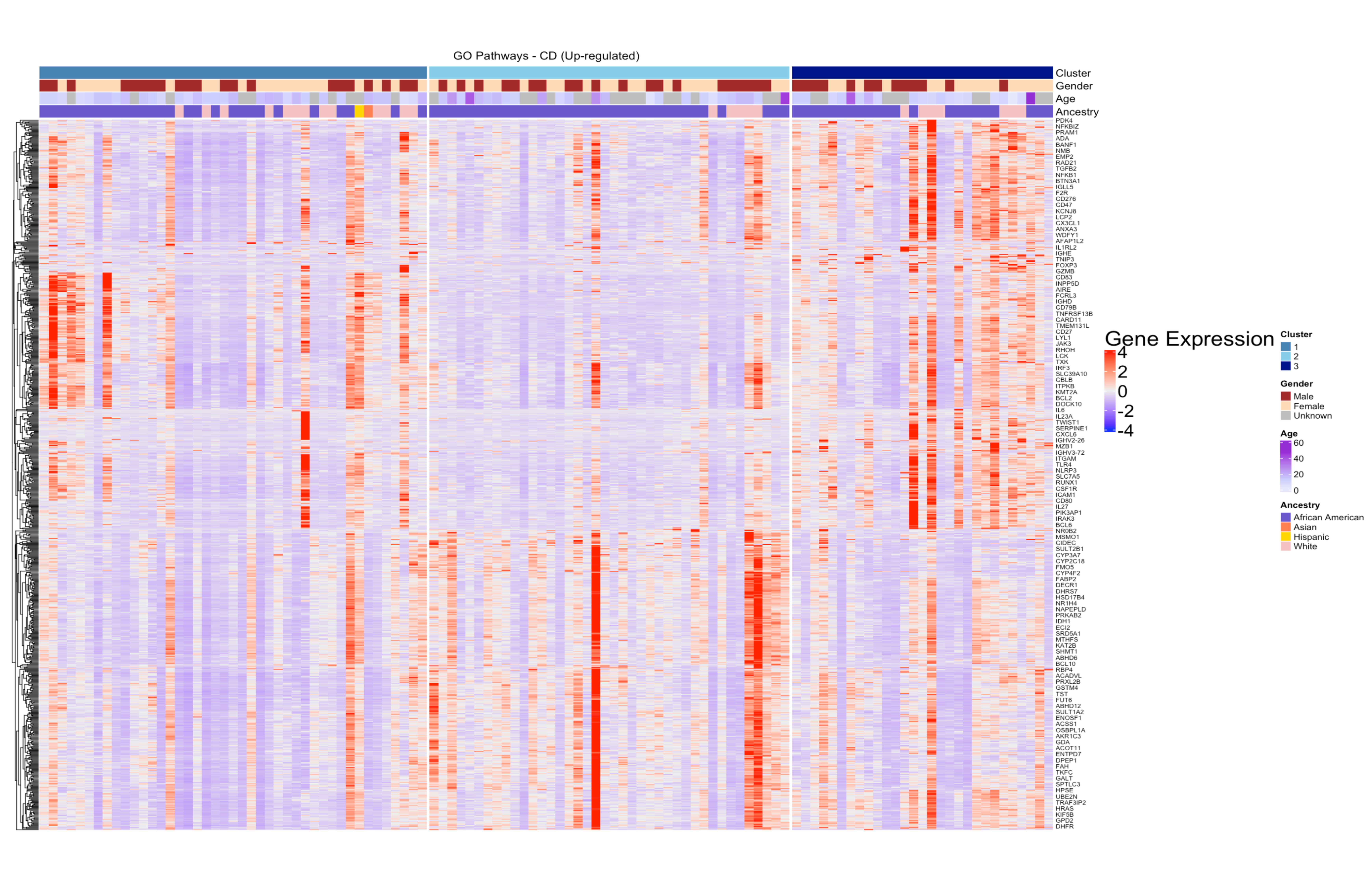


Figure S17: Heatmap of Genes from the Top 5 Up-regulated GO Pathways. and down regulated GO pathways. This heatmap visualizes the expression levels of genes from the top 5 downregulated GO pathways in CD clusters within the GSE137344 validation dataset. Samples are annotated by clusters and clinical features, including gender, age, and ancestry. The plot highlights the differential expression patterns of genes across clusters, emphasizing their roles in up and down regulated GO pathways.


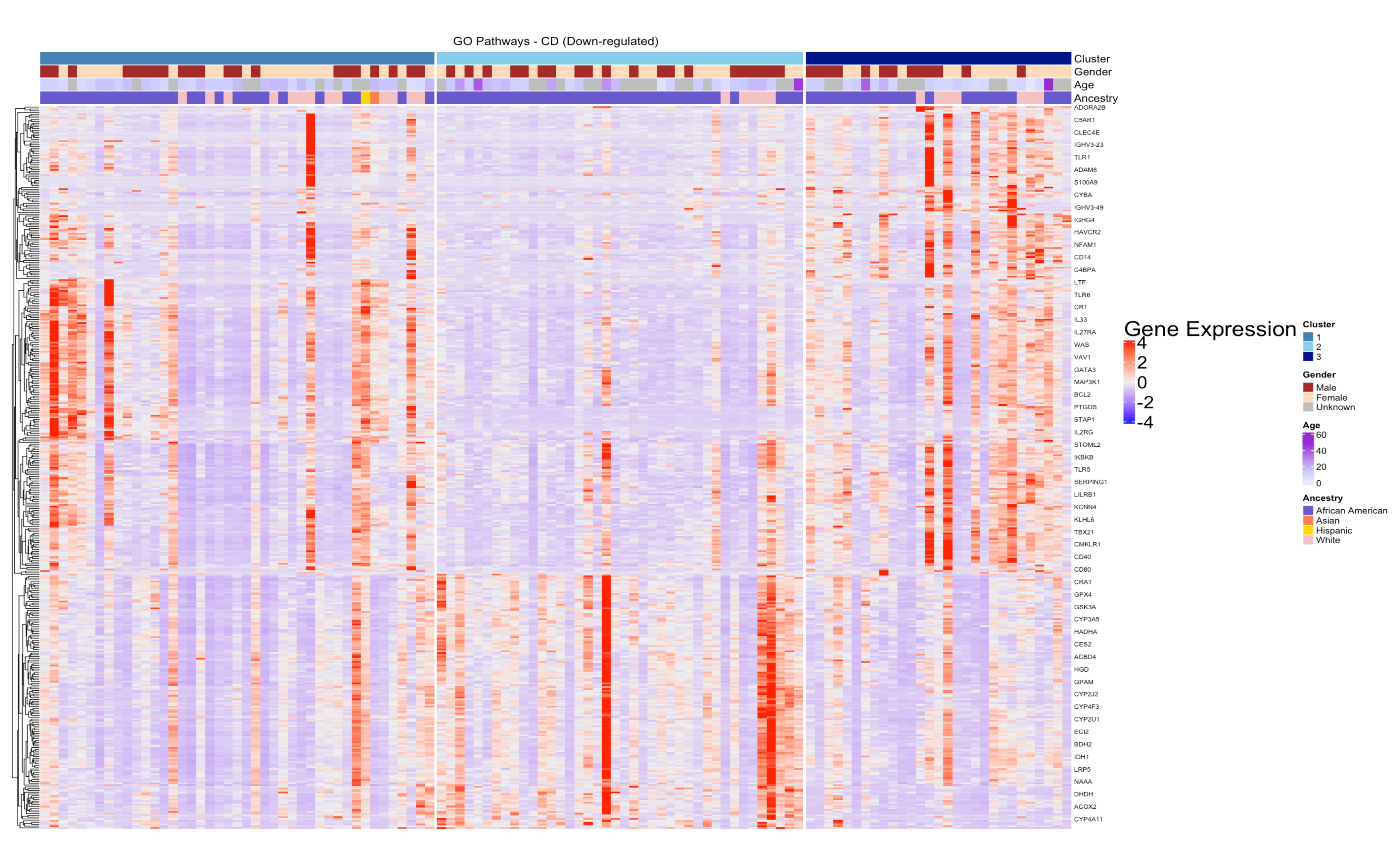


Figure S18: Heatmap of Genes from the Top 5 Down-regulated GO Pathways. This heatmap visualizes the expression levels of genes from the top 5 downregulated GO pathways in CD clusters within the GSE137344 validation dataset. Samples are annotated by clusters and clinical features, including gender, age, and ancestry. The plot highlights the differential expression patterns of genes across clusters, emphasizing their roles in up and down regulated GO pathways.


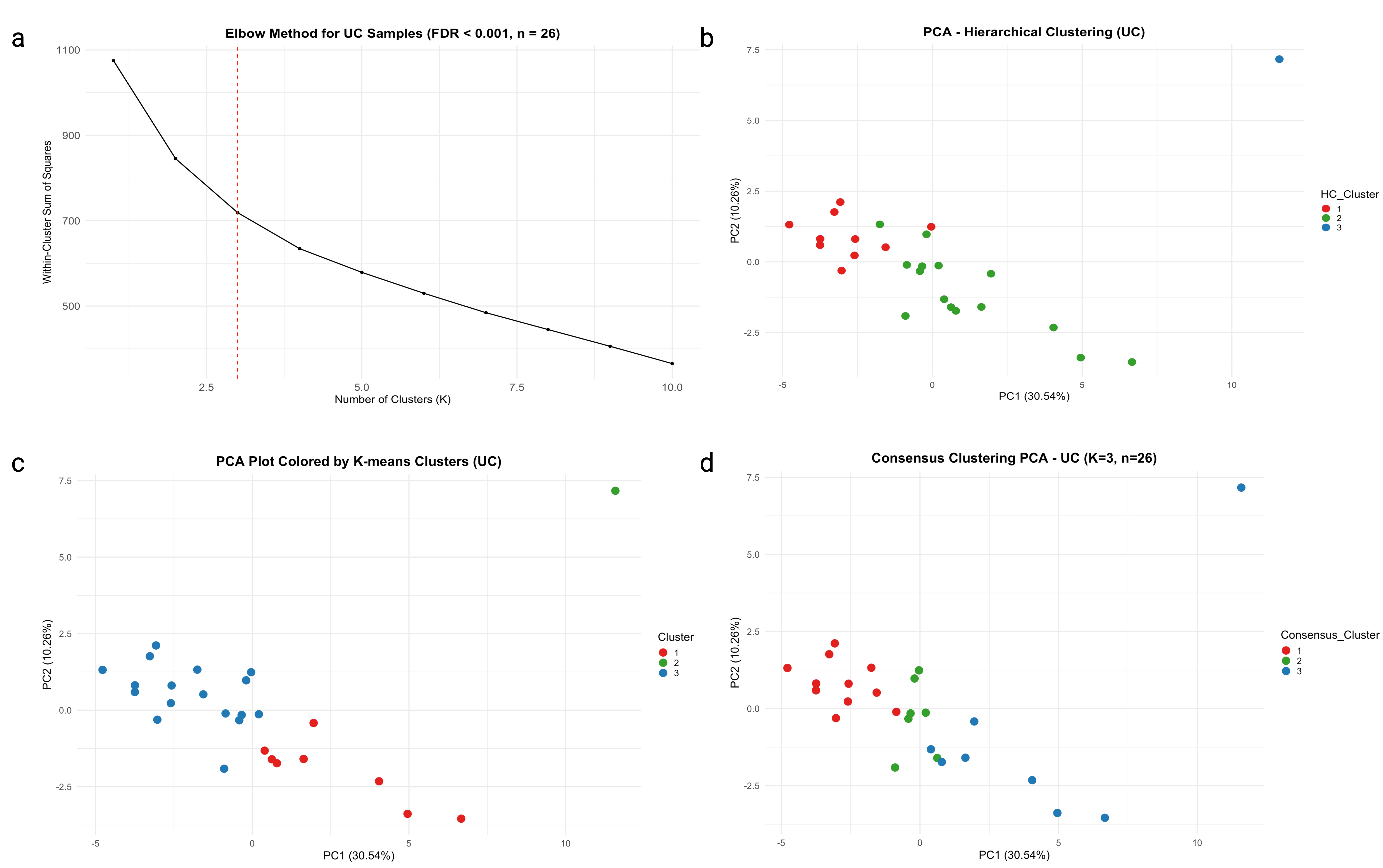


Figure S19: Results from K value selection and clustering analysis on GSE235236 UC validation dataset. a. Elbow plot showing k=3 as appropriate K- value. b,c,d. PCA Plot of Hierarchical, K-means and Consensus Clustering showing 3 clusters within UC samples respectively. a. X- axis represents the number of clusters (k) and, Y- axis depicts Within-cluster sum of squares (WCSS) and the red dotted line indicates the optimum value of k. b,c,d. X-axis and Y-axis shows PC1 and PC2 respectively with variance in %.
